# Clinical epidemiology and case fatality due to antimicrobial resistance in Germany: a systematic review and meta-analysis, 1 January 2010 to 31 December 2021

**DOI:** 10.2807/1560-7917.ES.2023.28.20.2200672

**Published:** 2023-05-18

**Authors:** Maria Rödenbeck, Olaniyi Ayobami, Tim Eckmanns, Mathias W Pletz, Jutta Bleidorn, Robby Markwart

**Affiliations:** 1 Institute of General Practice and Family Medicine, Jena University Hospital, Friedrich Schiller University Jena, Jena, Germany; 2 Unit for Healthcare Associated Infections, Surveillance of Antimicrobial Resistance and Consumption, Department of Infectious Disease Epidemiology, Robert Koch Institute, Berlin, Germany; 3 Institute for Infectious Diseases and Infection Control, Jena University Hospital, Friedrich Schiller University Jena, Jena, Germany; 4 InfectoGnostics Research Campus Jena, Jena, Germany

**Keywords:** Antimicrobial resistance, Antibiotic resistance, Germany, ESKAPE, Case fatality

## Abstract

**Background:**

Antimicrobial resistance (AMR) is of public health concern worldwide.

**Aim:**

We aimed to summarise the German AMR situation for clinicians and microbiologists.

**Methods:**

We conducted a systematic review and meta-analysis of 60 published studies and data from the German *Antibiotic-Resistance-Surveillance* (ARS). Primary outcomes were AMR proportions in bacterial isolates from infected patients in Germany (2016–2021) and the case fatality rates (2010–2021). Random and fixed (common) effect models were used to calculate pooled proportions and pooled case fatality odds ratios, respectively.

**Results:**

The pooled proportion of meticillin resistance in *Staphylococcus aureus* infections (MRSA) was 7.9% with a declining trend between 2014 and 2020 (odds ratio (OR) = 0.89; 95% CI: 0.886–0.891; p < 0.0001), while vancomycin resistance in *Enterococcus faecium* (VRE) bloodstream infections increased (OR = 1.18; (95% CI: 1.16–1.21); p < 0.0001) with a pooled proportion of 34.9%. Case fatality rates for MRSA and VRE were higher than for their susceptible strains (OR = 2.29; 95% CI: 1.91–2.75 and 1.69; 95% CI: 1.22–2.33, respectively). Carbapenem resistance in Gram-negative pathogens (*Klebsiella pneumoniae*, *Acinetobacter baumannii*, *Enterobacter* spp. and *Escherichia coli*) was low to moderate (< 9%), but resistance against third-generation cephalosporins and fluoroquinolones was moderate to high (5–25%). *Pseudomonas aeruginosa* exhibited high resistance against carbapenems (17.0%; 95% CI: 11.9–22.8), third-generation cephalosporins (10.1%; 95% CI: 6.6–14.2) and fluoroquinolones (24.9%; 95% CI: 19.3–30.9). Statistical heterogeneity was high (I^2^ > 70%) across studies reporting resistance proportions.

**Conclusion:**

Continuous efforts in AMR surveillance and infection prevention and control as well as antibiotic stewardship are needed to limit the spread of AMR in Germany.

## Introduction

The increasing occurrence of antimicrobial resistance (AMR) in bacterial infections has emerged as one of the biggest threats to global health [1]. Recent studies estimated that AMR was associated with 1.27 million attributable deaths worldwide in 2019 [2] and more than 33,000 deaths in the European Union and European Economic Area (EU/EEA) in 2015 [3]. Although there is evidence that the prevalence of AMR is especially pronounced in low-income countries [4], high-income countries are also affected. In the EU/EEA, deaths due to infections with antibiotic-resistant bacteria increased 2.5-fold between 2007 and 2015 [3], and a rise in resistance proportions was observed in important bacteria, such as vancomycin-resistant *Enterococcus faecium* [5].

In Germany, measures to limit the spread of antibiotic resistance are bundled in the German antibiotic resistance strategy (DART) [6]. One element of DART is the surveillance of antibiotic resistance, which is implemented as the continuous national antibiotic resistance surveillance (ARS) of the Robert Koch Institute [7,8]. However, ARS lacks clinical data (e.g. case fatality rate or diagnoses), and the national representativeness depends on voluntarily participating laboratories, where coverage varies between German regions.

To our knowledge, no systematic summary of the epidemiology and case fatality rate of antibiotic resistance in Germany has been published. However, recent and comprehensive AMR data are important to develop evidence-based treatment guidelines. We therefore conducted a systematic review and meta-analysis of published studies and data from the ARS database to analyse the national proportion of antibiotic resistance in relevant pathogens from clinical infections and the associated case fatality rate.

## Methods

This study followed the guidelines from the *Preferred Reporting Items for Systematic Reviews and Meta-Analyses* (PRISMA) statement [9]. The study protocol was published a priori in the Prospective Register for Systematic Reviews (PROSPERO, CRD42022306576) [10]. More details on methods are provided in the Supplementary material.

### Study outcomes

The primary outcomes of this study were (i) the antibiotic resistance proportion in bacterial isolates from infected patients in Germany and (ii) the attributable or all-cause case fatality rate of patients with infections caused by antibiotic-resistant bacteria. The antibiotic resistance proportion is defined as the total number of isolates tested as non-susceptible to a given antibiotic among all tested isolates. In order to study any potential change in antibiotic resistance proportions over the past years, we additionally performed time trend analyses using ARS data. Relevant pathogens and antibiotic resistance were based on the World Health Organization priority pathogens list for research and development of new antibiotics [11]. The included pathogens and bacteria are shown in the Box. We compared antibiotic resistance proportions from Germany with data from other regions/countries of the world (i.e. China, the EU/EEA, Japan, low- and lower-middle-income countries and the United States (US)).

Box **Pathogens and bacteria covered in this study**

**Pathogens:** *Acinetobacter baumannii, Pseudomonas aeruginosa, Escherichia coli, Enterobacter* spp*. (i.e. E. cloacae, E. aerogenes), Klebsiella pneumoniae, Enterococcus* spp. *(i.e. E. faecium, E. faecalis), Staphylococcus aureus, Helicobacter pylori, Campylobacter* spp., *Salmonella* spp., *Neisseria gonorrhoeae, Streptococcus pneumoniae, Haemophilus influenzae, Shigella* spp*., Clostridioides difficile.*

**Antibiotics:** Penicillins, ß-lactamase-stable penicillins (e.g. flucloxacillin, oxacillin, meticillin), penicillins and β-lactamase inhibitors, cephalosporins, carbapenems, fluoroquinolones, macrolides, glycopeptides, aminoglycosides, tetracyclines, clindamycin, cotrimoxazole, metronidazole, linezolid, daptomycin, colistin, rifampicin, fosfomycin, nitrofurantoin, trimethoprim, aztreonam, fusidic acid.

### Search strategy, study selection and data extraction

We conducted a systematic search in MEDLINE (PubMed) and Web of Science for studies on patients treated or diagnosed in Germany reporting the primary outcomes and published between 1 January 2016 and 31 December 2021 for AMR proportions and between 1 January 2010 and 31 December 2021 for case fatality data, using an a priori validated search string. Since our preliminary literature searches indicated that studies providing mortality data are scarce compared with studies providing AMR proportions, we extended the time period for the systematic literature search for studies in case fatality. Further, we included data from the ARS database [7] for AMR proportions (2019–2020) as well as time trend analyses and a comparison of outpatient vs inpatient setting (2014–2020). We also carried out a search in Google Scholar. 

Studies were included if they met the following criteria:

- The study reports at least one of the primary outcomes (resistance proportion or case fatality rate data for resistant pathogens);
- Publication period is 2010 to 2021 for studies reporting case fatality rate data and 2016 to 2021 for studies reporting resistance data;
- Data collection was completed after 2008 for case fatality rates and after 2015 for antibiotic resistance proportions;
- Data for the primary outcome ‘resistance proportion’ are provided for at least 20 clinical isolates;
- Language of publication: English or German;
- Phenotypic laboratory drug sensitivity testing was performed using defined cut-offs (breakpoints) based on accepted standards, such as those from the European Committee on Antimicrobial Susceptibility Testing (EUCAST) or the Clinical and Laboratory Standards Institute (CLSI);
- Germany is the place of sample/patient origin.

Studies that met the following criteria were excluded:

- Study design/type: editorials, case reports/case series reports, modelling studies, economic evaluations, reviews, duplicate records and studies reporting already published data, interventional studies (i.e. antibiotic effectiveness studies, studies on the effectiveness of antimicrobial stewardship and/or infection prevention and control measures), diagnostic accuracy studies (i.e. diagnostic accuracy studies on novel phenotypic and genotypic AMR diagnostics), studies reporting bacterial outbreaks only;
- Studies without quantitative data for the number of included isolates;
- Studied population: animals/plants only; patients only tested for colonisation or screening samples;
- Resistance was determined on genotypic level only (e.g. detection of resistance genes).

Two authors (MR, RM) performed title, abstract and full text screening as well as data extraction independently. Any discrepancies were resolved through discussion between MR and RM.

### Meta-analysis

Pooled estimates for antibiotic resistance proportions and case fatality rate (proportion of patients who died among all infected patients) were calculated using random effects models with a Freeman–Tukey Double Arcsine transformation of the raw proportions [12]. The pooled odds ratio of the case fatality rate between patients with antibiotic-resistant infections and patients with antibiotic-susceptible infections was calculated with a fixed (common) effect model for meta-analyses with binary outcome data using the Mantel–Haenszel method for pooling [13].

### Risk of bias assessment and statistical analyses

For studies reporting resistance proportions, we used the risk of bias assessment tool developed by Hoy et al. [14]. The Newcastle–Ottawa scale for cohort studies was used to assess the risk of bias in studies reporting case fatality data [15]. Meta-analyses were performed if at least three studies were included for a given outcome and pathogen–drug combination. All statistical analyses were performed using the software R Version 4.1.2 [16] and the ‘meta’ package [17,18].

## Results

**Figure 1:**
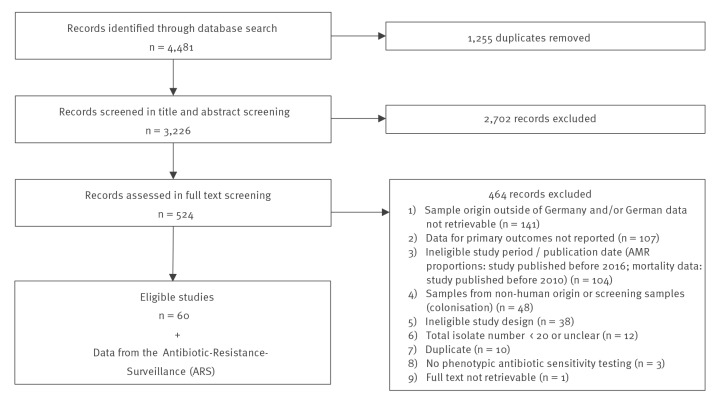
Flow chart of study selection on antimicrobial resistance, Germany, 2010–2021

Our systematic literature search yielded 3,226 unique records. After literature selection, 60 studies [19-78] and data from the ARS database were included in this study (Figure 1). In our study set, including ARS data, the most represented pathogens were from the ESKAPE-E group [79]: *Enterococcus* spp. (n = 11 *E. faecium*; n = 6 *E. faecalis*), *S. aureus* (n = 26), *K. pneumoniae* (n = 6), *A. baumannii* *complex* (n = 2), *P. aeruginosa* (n = 10), *Enterobacter* spp. (n = 2) and *E. coli* (n = 17). The study characteristics as well as a complete summary of all results are presented in Supplementary Tables S1–4 and Supplementary Figures S1–9.

AMR: antimicrobial resistance.

### Antibiotic resistance in Germany

The pooled proportion of meticillin-resistant *S. aureus* (MRSA) infections in Germany was 7.9% (95% confidence interval (CI): 5.2–11.0) (Table 1). Time trend analyses from ARS data showed that the proportions of MRSA among *S. aureus* decreased from 13.1% in 2014 to 6.7% in 2020 (odds ratio (OR) = 0.89; 95% CI: 0.88–0.89; p < 0.0001). No vancomycin or linezolid resistance was reported among clinical *S. aureus* isolates in Germany.

**Table 1:** Resistance proportions and time trend analysis of ESKAPE-E pathogens, Germany, 1 January 2016–31 December 2021

Resistance	Pooled resistance proportion % (95% CI)	Range of study estimates	Number of studies	Resistant isolates	Total isolates	Heterogeneity in % (I^2^ statistics)	ARS data^a^			
							Time trend		Odds ratio (95% CI)	Adjusted p value
*Enterococcus* spp.										
Vancomycin (VRE) in all infection types	15.8 (11.1–21.1)	0.0–52.2	15	4,003	26,321	98.6	NA			
Vancomycin (VRE) in BSI	23.3 (14.7-33.2)	0.0 - 52.2	7	295	1,102	76.4	↑↑	2015: 5.0% 2020: 9.9%	1.18 (1.16–1.22)	< 0.0001
Linezolid in all infection types	0.6 (0.0–3.2)	0.0–2.7	3	60	15,578	78.7	NA			
*Enterococcus faecium*										
Vancomycin (VREF) in all infection types	28.2 (23.9–32.7)	0.0–82.5	10	3,803	15,035	93.9	NA			
Vancomycin (VREF) in BSI	34.9 (25.2–45.4)	22.0 - 82.5	6	2,013	8,231	95.4	↑↑	2015: 11.9% 2020: 22.1%	1.18 (1.16–1.21)	< 0.0001^b^
Linezolid in BSI	Not pooled	0.6–2.7	2	49	7,551	NA	↔	2016: 0.04% 2020: 0.06%	1.03 (0.93–1.15)	0.488
*Enterococcus faecalis*										
Vancomycin	0.0 (0.0–0.0)	0.0–0.1	6	9	9,423	0	↔	2015: 0.1% 2020: 0.1%	1.0 (0.8–1.3)	0.805
Linezolid	Not pooled	0.1	1	9	9,423	NA	↔	2015: 0.02% 2020: 0.02%	0.84 (0.68–1.0)	0.0902
*Staphylococcus aureus*										
MRSA^b^	7.9 (5.2–11.0)	0.0–63.2	16	18,422	285,472	98.5	↓	2014: 13.1% 2020: 6.7%	0.89 (0.89–0.89)	< 0.0001
Vancomycin	0.0 (0.0–0.0)	0.0–0.0	4	0	416,849	0.0	↔	2014: 0.0% 2020: 0.0%	NA	
Linezolid	0.0 (0.0–0.0)	0.0–0.0	4	103	405,998	0.0	↔	2014: 0.1% 2020: 0.0%	0.74 (0.7–0.72)	1.0
*Klebsiella pneumonaie*										
Carbapenems	1.7 (0.0–5.9)	0.7–6.3	4	10,768	286,029	100.0	↔	2014: 1.2% 2020: 0.8%	0.98 (0.95–1.0)	1.0
Third-generation cephalosporins	10.7 (7.5–14.4)	7.4–14.3	3	22,612	226,825	96.2	↔	2014: 11.4% 2020: 9.5%	0.99 (0.98–0.99)	< 0.0001
Fluoroquinolones	15.5 (14.1–17.0)	13.3–16.4	3	13,509	82,486	69.2	↔	2014: 17.4% 2020: 15.8%	1.0 (1.0–1.01)	1.0
*Acinetobacter baumannii* (complex)										
Carbapenems	-	2.6–3.5	2	963	32,979	-	↓	2014: 5.2% 2020. 2.4%	0.91 (0.88–0.93)	< 0.0001
Fluoroquinolones	-	5.9	1	1,112	18,897	-	↓	2014: 12% 2020: 5.3%	0,88 (0.87–0.89)	< 0.0001
*Pseudomonas aeruginosa*										
Carbapenems	17.0 (11.9–22.8)	12.8–25.1	6	25,922	201,279	96.1	↔	2014: 13.1% 2020: 13.1%	1.0 (1.0–1.01)	1.0
Third-generation cephalosporins	10.1 (6.6 - 14.2)	4.7–15.5	5	311	2,515	81.5	↔	2014: 7.3% 2020: 8.2%	1.04 (1.03–1.04)	< 0.0001
Fluoroquinolones	24.9 (19.3–30.9)	15.8–33.3	6	25,598	85,825	95.3	↑	2014: 19.9% 2020: 35.4%	1.22 (1.22–1.23)	< 0.0001
*Enterobacter* spp.^c^										
Carbapenems	-	4.8–8.9	2	4,886	20,968	-	↑	2015: 6.8% 2020: 9.8%	1.12 (1.10–1.13)	< 0.0001
Third-generation cephalosporins	-	19.1–19.8	2	13,892	70,072	-	↔	2014: 23.4% 2020: 21.3%	0.97 (0.97–0.98)	< 0.0001
Fluoroquinolones	-	7.9–11.9	2	3,164	40,144	-	↔	2014: 8.2% 2020: 7.8%	1.0 (0.98–1.0)	1.0
*Escherichia coli*										
Carbapenems	0.0 (0.0–0.0)	0.0–0.1	10	616	619,515	0	↓	2015: 0.4% 2020: 0,1%	0.84 (0.82–0.87)	< 0.0001
Third-generation cephalosporins	11.1 (9.9–12.4)	7.1–19.2	13	133,059	1,464,011	99.2	↔	2014: 9.5% 2020: 8.3%,	0.99 (0.98–0.99)	< 0.0001
Fluoroquinolones	21.3 (19.9–22.8)	15.1–30.0	12	127,773	648,820	98.4	↔	2014: 19.9% 2020: 20.2%	1.02 (1.01–1.02)	< 0.0001

ARS: Antibiotic-Resistance-Surveillance database; BSI: bloodstream infections; CI: confidence interval; MRSA: meticillin-resistant *Staphylococcus aureus*; NA: not available; OR: odds ratio; VRE: vancomycin-resistant *Enterococcus* spp.; VREF: vancomycin-resistant *Enterococcus faecium.*

^a^ Time trend analyses are based on data from the ARS database (Robert Koch Institute) from in- and outpatients; p values are adjusted for multiple test using the Bonferroni method [96].

^b^ Resistance against β-lactamase-stable penicillins, such as meticillin, flucloxacillin and oxacillin.

^c^ In both studies, most *Enterobacter* species were *Enterobacter cloacae*: 90.5% in Dörr et al [23], 100% in ARS data.

In contrast to the development of MRSA, there was a major rise of vancomycin-resistant *E. faecium* (VRE) infections in the past decade in Germany. The VRE proportions in bloodstream infections (BSI) increased from 11.9% in 2015 to 22.1% in 2020 (OR = 1.18; 95% CI: 1.16–1.21; p < 0.0001), reaching a plateau in 2018 (23.6%). Pooled vancomycin resistance proportions in all infections were 15.8% (95% CI: 11.1–21.1) for *Enterococcus* spp. and 28.2% (95% CI: 23.9–32.7) for *E. faecium,* whereas pooled VRE proportions in BSI are as high as 34.9% (95% CI: 25.2–45.4) (Table 1). Vancomycin resistance was scarcely detected in clinical *E. faecalis* isolates in Germany (0–0.1%). Based on three studies, including national ARS data, resistance against linezolid, an important treatment alternative for VRE, was still low in *Enterococcus* spp. (0.6%; 95% CI: 0.0–3.2).

In clinical infections with Gram-negative organisms in Germany, proportions of carbapenem resistance were consistently low in *K. pneumoniae*, *A. baumannii* *complex* and *E. coli* isolates (< 3.5%), while the pooled proportion of carbapenem resistance in *P. aeruginosa* was as high as 17.0% (95% CI: 11.9–22.8; n = 6) (Table 1). A slight increase in carbapenem resistance (from 6.8% in 2015 to 9.8% in 2020) was found in *Enterobacter* spp. (OR = 1.12; 95% CI: 1.10–1.13; p < 0.0001).

Pooled resistance against third-generation cephalosporins was moderate (ca 10%) in *K. pneumoniae*, *P. aeruginosa* and *E. coli,* but high in *Enterobacter* spp. (19%), without significant changes over time in those pathogens. Pooled fluoroquinolone resistance was high in *K. pneumoniae* (15.5%; 95% CI: 14.1–17.0), *P. aeruginosa* (24.9%; 95% CI: 19.3–30.9) and *E. coli* (21.3%; 95% CI: 19.9–22.8) and moderate for *Enterobacter* spp. (7.9–11.9%). While fluoroquinolone resistance decreased between 2014 and 2020 in *A. baumannii complex* (12% in 2014 and 5.3% in 2020; OR = 0.88; 95% CI: 0.87–0.89; p < 0.0001), it increased in *P. aeruginosa* (2014: 19.9%, 2020: 35.4%; OR = 1.22; 95% CI: 1.22–1.23; p < 0.0001).

Notably, data from the ARS database indicated that antibiotic resistance proportions in all major pathogens were significantly higher in clinical isolates from inpatients compared to outpatients. Resistance proportions of inpatients vs outpatients for individual bacteria and antibiotics are appended in Supplementary Table S3 and Supplementary Figure S1. We found a high between-study heterogeneity (I^2^ > 75%) in almost all our analyses of pooled antibiotic resistance proportions.

### Case fatality rate

**Figure 2:**
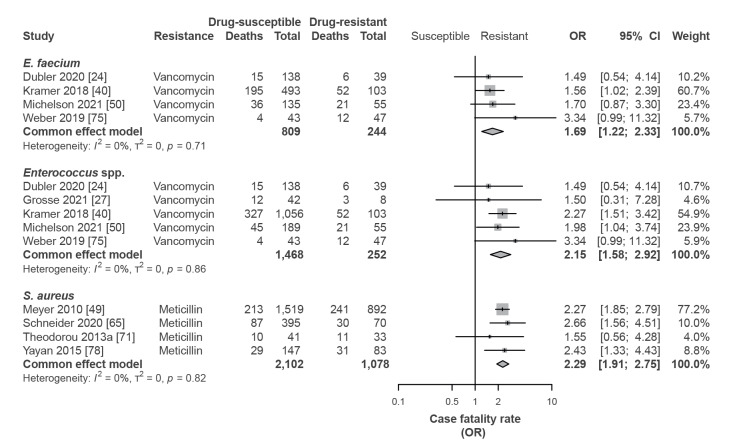
Case fatality rate of patients infected with vancomycin-resistant *Enterococcus* spp., vancomycin-resistant *E. faecium* and meticillin-resistant *Staphylococcus aureus* ^a^ compared with susceptible strains, Germany, 1 January 2010–31 December 2021

CI: confidence interval; OR: odds ratio.

^a^ Meticillin resistance includes resistance against β-lactamase-stable penicillins, such as meticillin, flucloxacillin and oxacillin.

Patients with infections with vancomycin-resistant *Enterococcus* spp*.* had a consistently higher case fatality rate than patients infected with vancomycin-susceptible enterococci (*Enterococcus* spp.: pooled OR = 2.15; 95% CI: 1.58–2.92; *E. faecium*: OR = 1.69; 95% CI: 1.22–2.33) (Figure 2). The pooled all-cause case fatality rate of patients infected with vancomycin-resistant *Enterococcus* spp*.* and *E. faecium* was 31.8% (95% CI: 21.9–42.6%) and 32.4% (95% CI: 17.9–48.8%), respectively. A complete summary of the data for case fatality rates is provided in Supplementary Table S4).

In line with the findings for enterococcal infections, the case fatality rate of patients with MRSA infections was higher than of patients with meticillin-sensitive *S. aureus* infections (pooled OR = 2.29; 95% CI: 1.91–2.75) (Figure 2). The pooled all-cause case fatality rate of patients infected with MRSA was 28.5% (95% CI: 20.0–37.9) (data appended in full in Supplementary Table S4). For extended spectrum β-lactamase (ESBL)-positive *E. coli*, two studies reported case fatality rates of 25.3% and 23.8%, while for ESBL-negative *E. coli,* it was 17.6% in one study [46,63]. In contrast, ESBL status in *K. pneumoniae* did not vary with case fatality rates in two studies (ESBL-positive: 24.2% and 27.1%; ESBL-negative 25.2%) [46,63]. In *P. aeruginosa* bacteraemia, one study [77] reported that the case fatality rate was significantly higher in MDR isolates (EBSL and/or carbapenem resistance) compared with non-3/4 MDR isolates (63 vs 30%), while another study [72] did not find any difference (42 vs 45%) (data appended in full in Supplementary Table S4).

### Risk of bias

For studies reporting AMR proportions, there was an overall low risk of bias for the internal validity in 36 of the 44 included studies. In the Supplementary material, pages 6–11, we provide the detailed results of the risk of bias assessment. However, none of the studies met or clearly indicted national representativeness. For studies reporting case fatality data, the risk of bias yielded scores between five and seven of nine possible points, indicating a moderate to high quality of the included studies. Again, representativeness of the included cases was unclear in 19 of 20 studies.

### Comparison of resistance proportions in ESKAPE-E organisms between Germany and other countries/regions

Infections with MRSA have steadily decreased in Germany over the past years, and resistance proportions are low compared with other countries (Table 2). In comparison with the EU/EEA average (16.7%) and with Japan (1.6%), vancomycin resistance in clinical *E. faecium* isolates from in Germany is much higher, but still lower than the estimates from the US. Compared with the EU/EEA, China, low- and lower-middle-income countries and the US, *K. pneumonai*e and *A. baumannii* in Germany had low proportions of resistance to carbapenems, third-generation cephalosporins and fluoroquinolones. On the other hand, resistance proportions in *P. aeruginosa* in Germany were moderate to high and comparable to other countries in the world. While carbapenem resistance proportions in clinical *E. coli* isolates were rare in China, EU/EEA, Germany, Japan and the US, carbapenem resistance in *E. coli* was very frequent in low- and lower-middle-income countries. Fluoroquinolone resistance in *E. coli* was high (> 21%) in all countries addressed in Table 2.

**Table 2:** Comparison of resistance proportions in ESKAPE-E organisms between Germany and other countries/regions, 1 January 2016–31 December 2021

Pathogen/resistance	Germany^a^	EU/EEA^b^	United States^c^	Low-/lower-middle-income countries^d^	Japan^e^	China^f^
***Staphylococcus aureus***						
Meticillin^g^	7.9%	16.7%	40.6%	48.2%	47.6%	NA
***Enterococcus faecium***						
Vancomycin	28.2%	16.8%	65.7%	NA	1.6%	NA
***Klebsiella pneumoniae***						
Carbapenems	1.7%	10.0%	4.8%	34.8%	0.5%	20.9%
Third-generation cephalosporins	10.7%	33.9%	24.4%	78.7%	11.4%	47.3%
Fluoroquinolones	15.5%	33.8%	NA	NA	5.6%	32.2%
***Acinetobacter baumannii* (complex)**						
Carbapenems	2.6–3.5%	38.0%	40.0%	72.4%	1.8%	70.7%
Fluoroquinolones	5.9%	41.8%	NA	NA	12.7%	46.8%
***Pseudomonas aeruginosa***						
Carbapenems	17.0%	17.8%	12.9%	37.1%	20%	23.6%
Third-generation cephalosporins	10.1%	NA	16.0%	NA	14.1%	21.4%
Fluoroquinolones	24.9%	19.6%	15.2%	NA	15.3%	14.8%
***Enterobacter* spp.**						
Carbapenems	4.8-8.9%	NA	5.7%	51.2%	4.6%	NA
Third-generation cephalosporins	19.1–19.8%	NA	NA	83.5%	37.2%	NA
Fluoroquinolones	7.9–11.9%	NA	NA	NA	5.2%	NA
***Escherichia coli***						
Carbapenems	0.0%	0.2%	0.8%	16.6%	0.2%	1.9%
Third-generation cephalosporins	11.1%	14.9%	24.7%	78.6%	28.9%	59.3%
Fluoroquinolones	21.3%	23.8%	35.2%	NA	43.5%	57.0%

EEA: European Economic Area; EU: European Union, NA: not available.

^a^ Pooled resistance proportions of primary studies and ARS data from this study.

^b^ Source: *Antimicrobial resistance in the EU/EEA (EARS-Net) Annual Epidemiological Report for 2020 [* 83 *]: 29* EU/EEA countries reported data for 2020 to the European Antimicrobial Resistance Surveillance Network (EARS-Net). EARS-Net includes data for isolates from invasive infections (cerebrospinal fluid + bloodstream). *Acinetobacter* is not differentiated to species level.

^c^ Source: Centers for Disease Control and Prevention *National Healthcare Safety Network* (NHSN) 2020, Antibiotic Resistance & Patient Safety Portal [97], nationwide surveillance network for healthcare-associated infections, including ca. 25,000 medical facilities. *Acinetobacter* and *Klebsiella* not differentiated to species level.

^d^ Source: Data from 2010 to 2020 in a systematic review and meta-analysis of 163 studies on antibiotic resistance in hospital-acquired ESKAPE-E infections in low- and lower-middle-income countries [4].

^e^ Source: *Japan Nosocomial Infections Surveillance* (JANIS) 2020 [98], comprehensive nationwide surveillance programme with ca. 2,000 participating hospitals funded by the Japanese Ministry of Health, Labour and Welfare and managed by the National Institute of Infectious Diseases. *Acinetobacter* not differentiated to species level.

^f^ Source: *China Antimicrobial Surveillance Network* (CHINET) in 2017 [99]. CHINET was established in 2005 to gather nationwide data of the prevalence of bacteria and changes in rates of antimicrobial resistance. The CHINET surveillance system covers more than half of the Chinese population. *Acinetobacter* not differentiated to species level.

^g^ Resistance against β-lactamase-stable penicillins such as meticillin, flucloxacillin and oxacillin.

## Discussion

Based on the analysis of 60 primary studies and surveillance data from the ARS database, our systematic review provides a comprehensive summary of the antibiotic resistance situation of important pathogens and their associated case fatality rate in Germany.

Our study shows a marked increase of vancomycin resistance in clinical *E. faecium* isolates in Germany between 2010 and 2021, with pooled resistance proportions as high as 34.9% in BSI. In addition, there was a major increase in cases of vancomycin resistant *E. faecium* in German hospitals between 2014 and 2017 [47]. This is of public health concern because VRE infections are associated with a significantly higher mortality and economic burden than infections with vancomycin-sensitive strains [80]. Although increasing VRE proportions were observed in all European regions, recent proportions in Germany were higher than in neighbouring countries (e.g. Austria, Denmark, France and the Netherlands) except Czechia and Poland [5]. The reasons for the rise in VRE in Germany are largely unknown, but there is no routine VRE screening in German hospitals and official hygiene recommendations only focus on the prevention of VRE infections that require antibiotic therapy [81]. Fortunately, resistance to linezolid, an important treatment alternative for VRE, is still very low in enterococcal infections in Germany and in Europe [82]. However, there is evidence from the Jena University Hospital and other hospitals (data not shown) that linezolid resistance is emerging, which calls for increased attention and surveillance.

In contrast, we observed that MRSA infections have steadily decreased in Germany over the past years and resistance proportions are now low compared to southern (e.g. Italy and Spain) and eastern (e.g. Bulgaria and Poland) European countries [83]. However, MRSA proportions in Germany are still much higher than in Scandinavian countries where resistance proportions are below 5%. This decline of MRSA in Germany might be associated with the implementation of improved national infection prevention and control strategies (IPC) from 2014 onwards [84], which correlated with the decline in the MRSA proportion in *S. aureus* (a graphic visualisation can be found in Supplementary Figure S3)*.* These IPC measures include routine MRSA screening, mandatory reporting, appropriate isolation and the availability of MRSA eradication for colonised patients that are now routinely used in German hospitals [85,86]. Nonetheless, MRSA infections still represent a significant burden for German healthcare and are associated with increased case fatality rates compared with meticillin-sensitive *S. aureus* infections.

Carbapenem resistance in infections with Gram-negative pathogens was low and is much lower in Germany than in low- and lower-middle-income countries. However, in clinical *P. aereuginosa* isolates from Germany, carbapenem resistance proportions of 17% were observed, which is similar to other large European countries, such as Austria, France, Italy and Spain. Resistance against third-generation cephalosporins in *K. pneumoniae*, *P. aeruginosa* and *E. coli* infections remained stable on a moderate level around 10% in Germany. Only Scandinavian and Benelux countries tend to have lower third-generation cephalosporin resistance levels in Gram-negative bacterial isolates [83]. Fluoroquinolone resistance in infections with *K. pneumoniae, P. aeruginosa* and *E. coli* was high in Germany (> 15%) with an increasing trend in *P. aeruginosa*. The AMR situation of *P. aeruginosa* is of concern, as it is a common cause of nosocomial pneumonia, chronic wound and urinary tract infections and a risk for patients with compromised immune systems [87].

The higher resistance proportions among inpatients compared with outpatients is not surprising because hospital patients are at higher risk of acquiring nosocomial infections and antibiotic prophylaxis and treatment often include multiple drugs at higher doses and for longer periods than in outpatient practices, which promotes the development of AMR [88]. Moreover, outpatient antibiotic prescriptions have consistently been decreasing in Germany [89].

Antibiotic resistance proportions in the studied pathogens were generally lower in Germany than in low- and lower-middle-income countries. This finding is line with data from *the Global Burden of Disease Study 2019*, which showed that the burden of infections with antibiotic-resistant bacteria is much higher in global areas with limited resources (e.g. sub-Saharan countries) than in high-income areas such as western Europe and North America [90].

Similar to other reviews on antibiotic resistance patterns [4,91], we found a large variance across individual study estimates of AMR proportions. The emergence and spread of AMR are influenced by multiple factors including microbiological, environmental as well as societal and economic factors, with regional and local peculiarities [92]. In addition, variability in study settings (e.g. local IPC and antibiotic stewardship measures), sample selection as well as differences in pathogen identification and antimicrobial susceptibility testing method may explain the large heterogeneity.

To our knowledge, our study represents the first systematic review of resistance proportions and case fatality rates of infections with major pathogens in Germany. A particular strength of our study is that we included more comprehensive data sources by combining data from the German national AMR surveillance (ARS) with data from 60 primary studies. However, there are some limitations in our study. Representativeness of the included datasets was unclear but there were no differences between studies providing regional data vs. studies with national coverage (see detailed results in Supplementary Table S5). AMR proportions differed significantly across individual studies, therefore the pooled AMR proportions must be interpreted with caution. Importantly, EUCAST redefined the susceptibility testing categories (*susceptible* (S), *susceptible, increased exposure* (I), *resistant* (R)) in 2019 [93]. Since most studies included I and R in their resistance data, the EUCAST changes of the (I) category may have resulted in changed pooled resistance proportions compared with data from before 2019. Moreover, microbiological diagnostics are not recommended and routinely performed for typical infections, such as uncomplicated urinary tract infections in outpatient care, and therefore AMR patterns may not be adequately reflected in the data set [94]. Also, vancomycin-resistance proportions in *E. faecium* may be biased because enterococci are often only differentiated into species level if susceptibility testing reveals resistance to vancomycin [95]. However, vancomycin resistance also increased in non-differentiated *Enterococcus* spp. isolates [58]. In addition, the composition of clinical samples in ARS varies with changes in participating laboratories, which may have an impact on time trend analyses [95]. Another limitation is that our literature search for individual studies only included two databases (and manual search) and we did not perform a systematic search in the references of retrieved studies. Moreover, few German studies provided data on case fatality associated with infections caused by antibiotic-resistant Gram-negative bacteria, which should be investigated in future studies.

## Conclusion

Although antibiotic resistance in major bacterial pathogens is often less prevalent in Germany than in other countries (especially compared to countries with limited resources), worrying patterns and trends of resistance against important antibiotics are observed, especially in patients treated in hospitals. Continuous efforts in IPC as well as antibiotic stewardship are needed to limit the spread of AMR in Germany. Moreover, improved national AMR surveillance and well-designed studies with nationally representative data, including clinical outcomes, are important to provide data for evidence-based treatment and IPC guidelines.

## Supplementary Data

1990000 Supplement
