# Supplementary material for: Clinical epidemiology and case fatality due to antimicrobial resistance in Germany: a systematic review and meta-analysis, 1 January 2010 to 31 December 2021
Source: Euro Surveill. 2023 May 18;28(20):2200672. doi: 10.2807/1560-7917.ES.2023.28.20.2200672 (PMC10197495; doi:10.2807/1560-7917.ES.2023.28.20.2200672)
Supplement: Supplement [file 22-00672_MARKWART_Supplement.pdf]

This supplementary material is hosted by *Eurosurveillance* as supporting information alongside the article "Clinical epidemiology and case fatality due to antimicrobial resistance in Germany: a systematic review and meta-analysis, 1 January 2010 to 7 January 2022", on behalf of the authors, who remain responsible for the accuracy and appropriateness of the content. The same standards for ethics, copyright, attributions and permissions as for the article apply. Supplements are not edited by *Eurosurveillance* and the journal is not responsible for the maintenance of any links or email addresses provided therein.

- Supplementary Material -

Clinical epidemiology and case fatality due to antimicrobial resistance in Germany: a systematic review and meta-analysis, 1 January 2010 to 31 December 2021

Contents

1. Search strings ... 2
2. Study selection and inclusion / exclusion criteria ... 4
3. Data extraction ... 4
4. Statistical analyses ... 5
5. Risk of bias assessment ... 6
6. Study characteristics ... 12
7. Results ... 16
7.1 Pooled resistance proportions of included pathogens ... 16
7.2 Subgroup-analysis: Outpatients vs. Inpatients ... 24
7.3 Time trend analysis ... 26
7.4 AMR proportions in different countries ... 32
7.5 Case fatality rate ... 33
7.6 Subgroup analyses of antimicrobial resistance proportions: studies with national data vs. studies with regional data. ... 34
8. References of the supplementary material ... 35

1. Search strings

1.1 MEDLINE (Pubmed)

Applied filters: *Publication date from 2010 - 2021*
Search Date: *07.01.2022*
Number of results: ***1919***

("Acinetobacter baumannii" OR "A. baumannii" OR "Acinetobacter baumannii" (Mesh) OR "Pseudomonas aeruginosa" OR "P. aeruginosa" OR "Pseudomonas aeruginosa" (Mesh) OR "Enterobacter*" OR "Enterobacter spp*" OR "Enterobacter species" OR "Enterobacter"(Mesh) OR "Enterobacter cloacae" OR "E. cloacae" OR "Enterobacter cloacae"(Mesh) OR "Enterobacter aerogenes" OR "E. aerogenes" OR "Enterobacter aerogenes"(Mesh) OR "Escherichia coli" OR "E. coli" OR "Escherichia coli" (Mesh) OR "CREC" OR "Klebsiella pneumoniae" OR "K. pneumoniae" OR "Klebsiella pneumoniae"(Mesh) OR "Enterococc*" OR "Enterococcus spp*" OR "Enterococcus species" OR "Enterococcus" (Mesh) OR "Enterococcus faecium" OR "E. faecium" OR "Enterococcus faecium"(Mesh) OR "Enterococcus faecalis" OR "E. faecalis" OR "Enterococcus faecalis"(Mesh) OR "VRE" OR "VREF" OR "Staphylococcus aureus" OR "S. aureus" OR "Staphylococcus aureus" (Mesh) OR "MRSA" OR "MSSA" OR "VRSA" OR "Helicobacter pylori" OR "H. pylori" OR "Helicobacter pylori" (Mesh) OR "Campylobacter*" OR "Campylobacter spp*" OR "Campylobacter species" OR "Campylobacter"(Mesh) OR "Salmonell*" OR "Salmonella spp*" OR "Salmonella species" OR "Salmonella" (Mesh) OR "Neisseria gonorrhoeae" OR "N. gonorrhoeae" OR "Neisseria gonorrhoeae"(Mesh) OR "Streptococcus pneumoniae" OR "S. pneumoniae" OR "Streptococcus pneumoniae"(Mesh) OR "Haemophilus influenzae" OR "H. influenzae" OR "Haemophilus influenzae"(Mesh) OR "Shigell*" OR "Shigella spp*" OR "Shigella species" OR "Shigella"(Mesh) OR "Clostridium difficile" OR "C. difficile" OR "Clostridioides difficile" OR "Clostridioides difficile"(Mesh))

AND

("resistan*" OR "drug resistan*" OR "antibiotic resistan*" OR "antimicrobial resistan*" OR "antibacterial resistan*" OR "Drug Resistance, Bacterial"(Mesh) OR "nonsusceptib*" OR "multidrug resistan*" OR "multiresistan*" OR "MDR" OR "XDR" OR "extensively drug resistan*" OR "PDR" OR "panresistan*" OR "pandrug resistan*" OR "Drug Resistance, Multiple, Bacterial"(Mesh) OR "susceptib*" OR "sensitiv*")

AND

("proportion*" OR "percent*" OR "epidemiolog*" OR "Epidemiological Monitoring" (Mesh) OR "Epidemiology"(Mesh) OR "epidemiology"(Subheading) OR "Antimicrobial Stewardship"(Mesh) OR "prevalen*" OR "Prevalence"(Mesh) OR "mortalit*" OR "Mortality"(Mesh) OR "lethalit*" OR "morbidit*" OR "Morbidity"(Mesh) OR "Surveillan*")

AND

("Germany"(Mesh) OR "german*" OR "Schleswig-Holstein" OR "mecklenburg-western pomerania" OR "lower saxony" OR "saxony-anhalt" OR "saxony" OR "north rhine-westphalia" OR "thuringia" OR "hesse" OR "rhineland-palatinate" OR "saarland" OR "baden-wurttemberg" OR "bavaria" OR "Berlin" OR "Hamburg" OR "Munich" OR "Cologne" OR "Frankfurt am Main" OR "Stuttgart" OR "Dusseldorf" OR "Leipzig" OR "Dortmund" OR "Essen" OR "Bremen" OR "Dresden" OR "Hanover" OR "Nuremberg" OR "Duisburg")

AND

("human*" OR "Humans"(Mesh) OR "patient*" OR "Patients"(Mesh) OR "nosocomial*" OR "hospital*" OR "Hospitals"(Mesh) OR "Inpatient*" OR "Inpatients"(Mesh) OR "outpatient*" OR "Outpatients"(Mesh) OR "ambulatory care" OR "Ambulatory Care"(Mesh) OR "emergency care" OR "Emergency Service, Hospital"(Mesh))

NOT

("veterinar*" OR "Veterinary Medicine"(Mesh))

1.2 Web of science

Applied filters: *Timespan 2010-01-01 to 2021-12-31 (Publication Date)*
Search Date: *07.01.2022*
Number of results: ***2562***

ALL=("Acinetobacter baumannii" OR "A. baumannii" OR "Pseudomonas aeruginosa" OR "P. aeruginosa" OR "Enterobacter*" OR "E. cloacae" OR "E. aerogenes" OR "Escherichia coli" OR "E. coli" OR "CREC" OR "Klebsiella pneumoniae" OR "K. pneumoniae" OR "Enterococc*" OR "E. faecium" OR "E. faecalis" OR "VRE" OR "VREF" OR "Staphylococcus aureus" OR "S. aureus" OR "MRSA" OR "MSSA" OR "VRSA" OR "Helicobacter pylori" OR "H. pylori" OR "Campylobacter*" OR "Salmonell*" OR "Neisseria gonorrhoeae" OR "N. gonorrhoeae" OR "Streptococcus pneumoniae" OR "S. pneumoniae" OR "Haemophilus influenzae" OR "H. influenzae" OR "Shigell*" OR "Clostridium difficile" OR "C. difficile" OR "Clostridioides difficile")

AND

ALL=("resistan*" OR "drug resistan*" OR "antibiotic resistan*" OR "antimicrobial resistan*" OR "antibacterial resistan*" OR "nonsusceptib*" OR "multidrug resistan*" OR "multiresistan*" OR "MDR" OR "XDR" OR "extensiv* drug resistan*" OR "PDR" OR "panresistan*" OR "pandrug resistan*" OR "susceptib*" OR "sensitiv*")

AND

ALL=("proportion*" OR "percent*" OR "epidemiolog*" OR "antimicrobial stewardship" OR "prevalen*" OR "mortalit*" OR "lethalit*" OR "morbidit*" OR "surveillan*")

AND

ALL=("german*" OR "Schleswig-Holstein" OR "mecklenburg-western pomerania" OR "lower saxony" OR "saxony-anhalt" OR "saxony" OR "north rhine-westphalia" OR "thuringia" OR "hesse" OR "rhineland-palatinate" OR "saarland" OR "baden-wurttemberg" OR "bavaria" OR "Berlin" OR "Hamburg" OR "Munich" OR "Cologne" OR "Frankfurt am Main" OR "Stuttgart" OR "Dusseldorf" OR "Leipzig" OR "Dortmund" OR "Essen" OR "Bremen" OR "Dresden" OR "Hanover" OR "Nuremberg" OR "Duisburg")

AND

ALL=("human*" OR "patient*" OR "nosocomial*" OR "hospital*" OR "inpatient*" OR "outpatient*" OR "ambulatory care" OR "emergency care")

NOT

ALL=("veterinar*")

2. Study selection and inclusion / exclusion criteria

Title, abstract and full text screening were performed independently by two review authors (MR, RM) using "Rayyan", a free software tool designed for performing systematic reviews (1). Any discrepancies were resolved through discussion.

3. Data extraction

Two reviewers (MR, RM) independently extracted the data of all eligible studies using a standard tabulator form in Microsoft Excel. All disagreements were resolved through discussion.

Following data were extracted:

Study title, study author, publication year, study year, study design and setting (single centre/multicentre/surveillance), study location (city, federal state), German region (Table S1), healthcare setting (in-/outpatients, healthcare ward), patient age (Table S2), disease and infection type (2), pathogen, antibiotic class and drug, number of resistant pathogens and total tested isolate number, antimicrobial susceptibility guideline, case fatality rate data, length of stay.

In cases where studies reported resistance data for several antibiotics from one antibiotic class (e.g. meropenem, imipenem, and ertapenem for carbapenem resistance), the data with the highest resistance proportion were extracted. The antibiotic resistance proportion is defined as the total number of isolates tested as resistant against a given antibiotic among all tested isolates as reported by the study. In studies that reported the number of resistant (R), intermediate (I) and susceptible (S), we extracted R+I as "resistant". From ARS data, we extracted only isolates that were tested as resistant (R).

Definition of German regions based on the German ARS database (3)

Region	Federal states
**North west**	Bremen, Hamburg, Lower-saxony, Schleswig-Holstein
**West**	North Rhine-Westphalia
**South west**	Baden-Württemberg, Hesse, Rhineland-Palatinate, Saarland
**South east**	Bavaria, Saxony, Thuringia
**North east**	Berlin, Brandenburg, Mecklenburg-Western Pomerania, Saxony-Anhalt

Definition of age groups:

**Category**	Neonates	Infants	Children	Adults	Elderly
**Patient Age**	< 1 month	< 12 months	1-18 years	> 18 years	> 65 years

4. Statistical analyses

All statistical analyses were performed using the software "R" Version 4.1.2 (4) and the "meta" package (5, 6). Meta-analyses were performed if at least 3 studies are included for a given outcome and pathogen-drug combination. I^2 statistics were used to quantify the statistical heterogeneity of the included studies. Additionally, time trend analyses of ARS data from 2014-2020 were performed using a binomial logistic regression model of the proportions adjusting for healthcare setting (i.e., outpatient care and inpatient care). In time trend analyses, we accounted for multiple testing by adjusting p-values using the Bonferroni method (7). We were not able to account for the varying participation in ARS over the years, but the number of participating laboratories and thus, the included hospitals / practices, remained relatively stable from 2016 onwards (https://ars.rki.de/Docs/Coverage.pdf [German]).

5. Risk of bias assessment

The risk of bias of all included studies was independently assessed by two reviewers (MR, RM). All disagreements were resolved through discussion.

In Order to assess the risk of bias for included studies reporting antibiotic resistance proportions, we used an adapted version of the "Risk of bias assessment checklist for prevalence studies" from Hoy et al. (8). This tool allows a judgment to the possible risk of bias on each included study which will be rated as "high risk" or "low risk". If there is insufficient detail reported in the study, we judged the risk of bias as "high risk".

Additionally, the Newcastle-Ottawa scale (NOS) (9) was used to assess the risk of bias in studies reporting case fatality rate data. In this review, we used the "NOS for cohort studies", defining cases as exposure to infection with antibiotic resistant pathogens. The NOS includes three domains of quality (i.e. selection, comparability, outcome) and rates each study from 0-9 stars.

Risk of bias assessment in the included studies reporting resistance proportions based on Hoy et al. (8)

	External Validity				Internal Validity				
	1. Was the study's target population and the sampling frame a close representation of the national population in relation to relevant variables, e.g. age, sex, occupation?	2. Was some form of random selection used to select the sample, OR, was a census undertaken?	3. Was the likelihood of non-response bias minimal?	4. Were data collected directly from the subjects (as opposed to a proxy)?	5. Was an acceptable case definition used in the study?	6. Was the study instrument that mea-sured the parameter of interest shown to have relia-bility and validity?	7. Was the same mode of data collection used for all subjects?	8. Was the length of the shortest prevalence period for the parameter of interest appropriate?	9. Were the numerator(s) and denominato r(s) for the parameter of interest appropriate?
Abdrabou 2021	High	High	Low	Low	Low	Low	Low	Low	Low
Banhart 2021	High	High	Low	Low	Low	Low	Low	Low	Low
Basha 2019	High	Low	Low	Low	Low	Low	Low	Low	Low
Doenhardt 2020	High	Low	Low	Low	Low	Low	Low	Low	Low
Doerr 2021	High	Low	Low	Low	Low	High	Low	Low	Low
Dubler 2020	High	Low	Low	Low	Low	Low	Low	Low	Low
Frickmann 2019	High	Low	Low	Low	Low	Low	Low	Low	Low
Friesen 2020	High	Low	Low	Low	Low	Low	Low	Low	Low
Große 2021	High	Low	Low	Low	Low	Low	Low	Low	Low
Grotelüschen 2019	High	Low	Low	Low	Low	Low	Low	Low	Low
Gudiol 2020	High	Low	Low	Low	Low	Low	Low	Low	Low
Hischebeth 2019	High	Low	Low	Low	Low	Low	Low	Low	Low
Hitzenbichler 2018	High	Low	Low	Low	Low	Low	Low	Low	Low
Hoppe 2018	High	Low	Low	Low	Low	Low	Low	Low	Low
Jarlier 2019	High	Low	Low	Low	Low	Low	Low	Low	Low
Klasan 2021	High	Low	Low	Low	Low	High	Low	Low	Low
Klein 2019	High	Low	Low	Low	Low	Low	Low	Low	Low
Klingeberg 2018	High	Low	Low	Low	Low	Low	Low	Low	Low

Koppe 2018	High	Low	Low	Low	Low	Low	Low	Low	Low
Köstlin-Gille 2021	High	Low	Low	Low	Low	High	Low	Low	Low
Kramer 2019a	High	Low	Low	Low	Low	High	Low	Low	Low
Kramer 2019b	High	Low	Low	Low	Low	High	Low	Low	Low
Kresken 2020	High	Low	Low	Low	Low	Low	Low	Low	Low
Markwart 2019	High	Low	Low	Low	Low	Low	Low	Low	Low
Meinen 2021	High	Low	Low	Low	Low	Low	Low	Low	Low
Michelson 2021	High	Low	Low	Low	Low	Low	Low	Low	Low
Nurjadi 2021	High	Low	Low	Low	Low	Low	Low	Low	Low
Nuernberg 2021	High	Low	Low	Low	Low	Low	Low	Low	Low
Olearo 2021	High	Low	Low	Low	Low	Low	Low	Low	Low
Perniciaro 2018	High	Low	Low	Low	Low	Low	Low	Low	Low
Pietsch 2021	High	Low	Low	Low	Low	Low	Low	Low	Low
Remschmidt 2018	High	Low	Low	Low	Low	Low	Low	Low	Low
Rothe 2019	High	Low	Low	Low	Low	Low	Low	Low	Low
Rupp 2021	High	Low	Low	Low	Low	Low	Low	Low	Low
Said 2021	High	Low	Low	Low	Low	Low	Low	Low	Low
Scheich 2018	High	Low	Low	Low	Low	High	Low	Low	Low
Schoeneweck 2021	High	Low	Low	Low	Low	Low	Low	Low	Low
Seitz 2017	High	Low	Low	Low	Low	Low	Low	Low	Low
Selb 2021	High	High	Low	Low	Low	Low	Low	Low	Low
Suwono 2021	High	Low	Low	Low	Low	Low	Low	Low	Low
Tessema 2021	High	Low	Low	Low	Low	Low	Low	Low	Low
Walker 2021	High	High	Low	Low	Low	High	Low	Low	Low
Weber 2019	High	Low	Low	Low	Low	High	Low	Low	Low
RKI (ARS)	High	Low	Low	Low	Low	Low	Low	Low	Low

Criteria for the answers:

1) The study’s target population and sampling frame was not (high risk)/was a close (low risk) representation of the national population.
2) A census was not undertaken (high risk)/was (low risk) undertaken or some form of random selection was used to select the sample (e.g. simple random sampling, stratified random sampling, cluster sampling, systematic sampling). For example, studies reporting data from national reference centers using preselected isolates were assessed with “high risk”.
3) The response rate was insufficient (high risk) or sufficient (low risk)
4) In some instances, data were collected from a proxy (high risk)/All data were collected directly from the subjects (low risk)
5) An acceptable case definition for infection was not (high risk)/was (low risk) used

6) The study instrument had not (high risk)/had been shown to have reliability and validity (pathogen identification and phenotypical drug sensitivity testing according to valid methods, e.g. EUCAST, CLSI)
7) The same mode of data collection was not (high risk)/was (low risk) used for all subjects
8) The shortest prevalence period for the parameter of interest was not (high risk)/was (low risk) appropriate
9) The paper did not (high risk)/did (low risk) present numerator(s) AND denominator(s) for the parameter of interest

Risk of bias assessment in the included studies reporting mortality / case fatality rate data based on the Newcastle-Ottawa Quality Assessment Scale for cohort studies (9)

A study can be awarded a maximum of one star for each numbered item within the *selection* and *outcome* categories. A maximum of two stars can be given for *comparability* (Item 5). For the quantification of a quality score, one point was given for each * achieved in the below checklist.

	**Selection**				**Comparability**	**Outcome**			
	1. Representa-tiveness of the exposed cohort	2. Selection of the non exposed cohort	3. Ascertain-ment of exposure	4. Demon-stration that outcome of interest was not present at start of study	5. Compara-bility of cohorts on the basis of the design or analysis	6. Assessment of outcome	7. Was follow-up long enough for outcomes to occur	8. Adequacy of follow up of cohorts	**Quality score**
Dubler, 2020		*	*	*	*	*	*	*	7/9
Große, 2021		*	*	*	*	*	*	*	7/9
Hos, 2017		NA	*	*	NA	*	*	*	5/7
Kramer, 2018		*	*	*	*	*	*	*	7/9
Lackermair, 2021		*	*	*	*	*	*	*	7/9
Leistner, 2014		*	*	*	*	*	*	*	7/9
Meyer, 2010		*	*	*	*	*	*	*	7/9
Michelson, 2021		*	*	*	*	*	*	*	7/9
Mutters, 2013		NA	*	*	NA	*	*	*	5/7
Neubeiser, 2019		NA	*	*	NA	*	*	*	5/7
Rhim, 2021		NA	*	*	NA	*	*	*	5/7
Sakellariou, 2016		NA	*	*	NA	*	*	*	5/7
Schneider, 2020		*	*	*	*	*	*	*	7/9
Theodorou, 2013a		*	*	*	*	*	*	*	7/9
Theodorou, 2013b		*	*	*	*	*	*	*	7/9
Walter, 2015	*	NA	*	*	NA	*	*	*	6/7
Weber, 2019		*	*	*	*	*	*	*	7/9
Wilke, 2017		NA	*	*	NA	*	*	*	5/7
Willmann, 2013		*	*	*	*	*	*	*	7/9
Yayan, 2015		*	*	*	*	*	*	*	7/9

Criteria for the answers:

1) There was a described truly or close representativeness of the national population (*)/there was a selected group/no description (no *).
2) The non-exposed (to infection with resistant pathogen) cohort was drawn from the same community as the exposed cohort (*) or was drawn from a different source/no description (no *). If there was no comparison reported, we awarded “NA”.
3) There was a reported record of exposure to antibiotic resistant pathogens (*) or no description (no *).
4) Demonstration that outcome of interest was not present at start of study.
5) There were study controls for “no infection” or “infection with susceptible pathogen” and the groups were in any (*)/all (**) additional factors comparable. If there was no control group reported, we awarded “NA”.
6) There was an independent blind assessment or a record linkage for the outcome (death) (*).
7) Follow-up was long enough for outcomes to occur (*).
8) There was a complete follow-up or a small number lost to follow-up (*).

6. Study characteristics

sTable 1: Study characteristics of included studies

Study	German region	Study design, data collection period	Patient types	Included bacterial isolates, number of isolates
Abdrabou, 2021	National	Surveillance, 2014-2019	Inpatients and outpatients (mixed ages), mixed infection types	*C. difficile*, N=1456
Banhart, 2021	National	Surveillance 2016-2018,	Inpatients and outpatients (mixed ages), mixed infection types	*N. gonorrhoeae*, N=1404
Basha, 2019	West	Single centre, 2016	Inpatients and outpatients (mixed ages), UTI	*E. coli*, N = 162 - 185
Dönhardt, 2020	South-East	Single centre, 2008-2018	Inpatients (infants), BSI/CSFI	*E. coli*, N = 73
Dörr, 2021	South-West	Single centre, 2018-2019	Inpatients (adults), SSTI	*E. coli*, N = 38,<br>*E. faecalis*, N = 32,<br>*E. faecium*, N = 4,<br>*Enterococcus* spp., N = 86,<br>*Enterobacter* spp., N = 42,<br>*P. aeruginosa*, N = 51,<br>*S. aureus*, N = 188
Dubler, 2020	South-West	Single centre, 2006-2016	Inpatients (adults), BSI/CSFI	*E. faecium*, N = 177
Frickmann, 2019	North-West	Single centre, Not reported	Inpatients (mixed ages), mixed infection types	*E. coli*, N = 336 - 975<br>*S. aureus*, N = 106 - 1035
Friesen, 2020	North-East	Multicentre, 2013-2017	Outpatients (mixed ages), SSTI	*S. aureus*, N = 483 - 592
Große, 2021	South-East, West	Multicentre 2009-2020	Inpatients (adults), IABI	*Enterococcus* spp., N = 51
Grotelüschen, 2019	North-West	Single centre, Not reported	Inpatients (adults), IABI	*E. coli*, N = 144,<br>*E. faecalis*, N = 48,<br>*E. faecium*, N = 58,<br>*Enterococcus* spp., N = 158
Gudiol, 2020	North-East, South-West	Multicentre, 2006-2018	Inpatients (adults), BSI/CSFI	*P. aeruginosa*, N = 41
Hischebeth, 2019	West	Single centre, not reported	Inpatients and outpatients (adults), BJI	*S. aureus*, N = 29
Hitzenbichler, 2018	South-East	Single centre, 2015-2017	Inpatients and outpatients (mixed ages), UTI	*E. coli*, N = 477
Hoppe, 2018	North-East	Single centre, 2012-2017	Inpatients and outpatients (children), SSTI	*S. aureus*, N = 50

Hos, 2017	West	Single centre, 2006-2012	Inpatients (adults), BSI/CSFI	*S. aureus*, N = 91
Jarlier, 2019	National	Surveillance, 2016	Inpatients and outpatients (mixed ages), BSI/CSFI	*E. coli*, N = 15619,<br>*K. pneumoniae*, N = 2809,<br>*P. aeruginosa*, N = 1320
Klasan, 2021	South-West	Single centre, 2006-2018	Inpatients (adults), BJI	*S. aureus*, N = 32
Klein, 2019	South-West	Multicentre, 2012-2016	Outpatients (mixed ages), SSTI	*S. aureus*, N = 2475
Klingeberg, 2018	National	Multicentre, 2015-2016	Outpatients (adults), UTI	*E. coli*, N = 598 - 631
Koppe, 2018	National	Surveillance, 2011-2016	Inpatients and outpatients (mixed ages), mixed infection types	*K. pneumoniae*, N = 56560 - 154524
Köstlin-Gille, 2021	National	Multicentre, 2009-2017	Inpatients (infants), BSI/CSFI	*E. coli*, N = 187,<br>*Enterococcus* spp., N = 136,<br>*S. aureus*, N = 239
Kramer, 2018	North-East	Multicentre, 2008-2015	Inpatients (mixed ages), BSI/CSFI	*E. faecium*, N = 103,<br>*Enterococcus* spp., N = 103
Kramer, 2019a	National	Surveillance, 2015-2016	Inpatients (mixed ages), mixed infection types	*S. aureus*, N = 553 - 2994
Kramer, 2019b	National	Multicentre, 2016	Inpatients (mixed ages), mixed infection types	*Enterococcus* spp., N = 44,<br>*S. aureus*, N = 19
Kresken, 2020	National	Multicentre, 2016-2017	Inpatients (mixed ages), mixed infection types	*P. aeruginosa*, N = 985
Lackermair, 2021	South-East	Single centre, 2012-2014	Inpatients (elderly), BJI	*S. aureus*, N = 24
Lâm, 2020	National	Surveillance, 2016	Inpatients (mixed ages), BSI/CSFI	*H. influenza*, N = 474
Leistner, 2014	North-East	Single centre, 2008-2011	Inpatients (adults), BSI/CSFI	*E. coli*, N = 1499,<br>*K. pneumoniae*, N = 352
Markwart, 2019	National	Surveillance, 2012-2017	Inpatients (mixed ages), mixed infection types	*E. faecium*, N = 6251
Meinen, 2021	National	Surveillance, 2012-2019	Inpatients and outpatients (mixed ages), DENT/ORAL	*S. aureus*, N = 2319 - 2345
Meyer, 2010	National	Surveillance, 2005-2009	Inpatients (mixed ages), PNEU	*S. aureus*, N = 2411
Michelson, 2021	South-East	Single centre, 2014-2016	Inpatients (mixed ages), BSI/CSFI	*E. faecalis*, N = 54,<br>*E. faecium*, N = 190<br>*Enterococcus* spp., N = 244
Mutters, 2013	South-West	Single centre, 2009-2010	Inpatients (mixed ages), BSI/CSFI	*Enterococcus* spp., N = 19
Neubeiser, 2019	National	Multicentre, 2016	Inpatients (mixed ages), mixed infection types	*Enterococcus* spp., N = 232,<br>S. aureus, N = 371
Nurjadi, 2021	South-West	Single centre, 2012-2019	Inpatients and outpatients (mixed ages), mixed infection types	*S. aureus*, N = 2319 - 2345
Nürnberg, 2021	National	Surveillance, 2016-2019	Inpatients and outpatients (mixed ages), BSI/CSFI	*H. influenza*, N = 2432

Olearo, 2021	North-West	Single centre, 2018	Inpatients (mixed ages), BSI/CSFI	*E. faecium*, N = 239
Perniciaro, 2018	National	Surveillance, 2016-2017	Inpatients and outpatients (children), BSI/CSFI	*S. pneumoniae*, N = 182
Pietsch, 2021	National	Surveillance, 2016-2019	Inpatients and outpatients (mixed ages), mixed infection types	*Salmonella* spp., N = 11730 - 13882
Remschmidt, 2018	National	Surveillance, 2016	Inpatients (mixed ages), mixed infection types	*Enterococcus* spp., N = 1550
Rhim, 2021	South-West	Single centre, 2003-2015	Inpatients (mixed ages), mixed infection type	*S. aureus*, N = 13
Rothe, 2019	South-East	Single centre, 2013-2018	Outpatients (mixed ages), BSI/CSFI	*Enterococcus* spp., N = 30, *P. aeruginosa*, N = 42, *S. aureus*, N = 92 *S. pneumoniae*, N = 34
Rupp, 2021	South-East	Single centre, 2017-2020	Inpatients (adults), BJI	*S. aureus*, N = 55
Said, 2021	National	Surveillance, 2014-2018	Inpatients and outpatients (mixed ages), mixed infection types	*A. baumannii*, N = 10918
Sakellariou, 2016	North-East	Single centre, 2008-2011	Inpatients (mixed ages), BSI/CSFI	*E. coli*, N = 160, *K. pneumoniae*, N = 59
Scheich, 2018	South-West	Single centre, 2008-2016	Inpatients (mixed ages), BSI/CSFI	*E. coli*, N = 65, *P. aeruginosa*, N = 22
Schneider, 2020	South-West	Single centre, 2012-2015	Inpatients (mixed ages), BSI/CSFI	*S. aureus*, N = 465
Schöneweck, 2021	South-East	Surveillance, 2018-2019	Inpatients (mixed ages), BSI/CSFI	*E. coli*, N = 518 - 1567, *E. faecium*, N = 48 - 186, *K. pneumoniae,* N = 92 - 270, *P. aeruginosa*, N = 120 - 131, *S. aureus*, N = 597 - 824
Seitz, 2017	South-East	Single centre, 2015-2017	Outpatients (adults), UTI	*E. coli*, N = 365
Selb, 2021	National	Surveillance, 2019-2021	Inpatients and outpatients (mixed ages), mixed infection types	*N. gonorrhoeae*, N = 145 - 468
Suwono, 2021	National	Surveillance, 2014-2017	Inpatients and outpatients (mixed ages), mixed infection types	*E. coli*, N = 324304
Tessema, 2021	South-East	Single centre, 2012-2020	Inpatients (neonates), BSI/CSFI	*E. coli*, N = 23
Theodorou, 2013a	West	Single centre, 1989-2009	Inpatients (mixed ages), BSI/CSFI	*S. aureus*, N = 74
Theodorou, 2013b	West	Single centre, 1989-2009	Inpatients (mixed ages), BSI/CSFI	*P. aeruginosa*, N = 87
Walker, 2021	National	Surveillance, 2018-2019	Not reported, BSI/CSFI	*E. faecalis*, N = 853, *E. faecium*, N = 491, *Enterococcus* spp., N = 1344

Walter, 2015	National	Surveillance, 2014	Inpatients and outpatients (mixed ages), BSI/CSFI	*S. aureus*, N = 3662
Weber, 2019	South-West	Single centre, 2007-2017	Inpatients (adults), BSI/CSFI	*E. faecalis*, N = 33,<br>*E. faecium*, N = 57,<br>*Enterococcus* spp., N = 90
Wilke, 2017	National	Multicentre, 2008-2012	Inpatients (adults), PNEU	*S. aureus*, N = 226
Willmann, 2013	South-West	Multicentre, 2006-2012	Inpatients (adults), BSI/CSFI	*P. aeruginosa*, N = 113
Yayan, 2015	West	Single centre, 2004-2014	Inpatients (mixed ages), PNEU	*S. aureus*, N = 230
Robert Koch-Institute	National	Surveillance 2019-2020	Inpatients and outpatients (mixed ages), mixed infection types	*A. baumannii,* N = 8782 - 24618<br>*E. coli*, N = 187823 - 1227380,<br>*E. faecalis*, N = 995 - 8353,<br>*E. faecium*, N = 3514 - 7382,<br>*Enterococcus* spp., N = 4509 - 15735,<br>*Enterobacter* spp., N = 20926 - 102973,<br>*K. pneumoniae*, N = 43820 - 238992,<br>*P. aeruginosa*, N = 83317 - 204445,<br>*S. aureus*, N = 9305 - 475980<br>*S. pneumoniae*, N = 1693 - 20415

Abbreviation of infection types: UTI = urinary tract infection, BSI/CSFI = bloodstream infection/cerebrospinal fluid infection, SSTI = soft skin and tissue infection, IABI = intraabdominal infection, BJI = bone and joint infection, DENT/ORAL = dental and oro-maxillofacial infections, PNEU = pneumonia.

The definition of infection types is based on the CDC/NHSN Surveillance Definitions for Specific Types of Infections (2).

7. Results

7.1 Pooled resistance proportions of included pathogens

sTable 2:

Pathogen and antibiotic resistance	Pooled resistance proportion in % (95% CI)	No. of studies, No. of resistant isolates / No. of total isolates	Range of individual study estimates in %	Heterogeneity in % (95% CI, p-value)
***A. baumannii* (complex)**				
Aminoglycosides	**-**	n = 1,<br>523 / 8782	**6.0**	-
Carbapenems	**-**	n = 2,<br>1: 380 / 10918,<br>2: 583 / 22061	**2.6 – 3.5**	-
Cotrimoxazole	**-**	n = 1,<br>829 / 24618	**3.3**	-
Fluoroquinolones	**-**	n = 1,<br>1112 / 18897	**5.9**	-
C. difficile				
Fluoroquinolones	**-**	n = 1 ,<br>831 / 1456	**57.1**	-
Glycopeptides	**-**	n = 1,<br>0 / 1456	**0.0**	-
Macrolides	**-**	n = 1,<br>775 / 1456	**53.2**	-
Metronidazole	**-**	n = 1,<br>39 / 1456	**2.7**	-
Rifampicin	**-**	n = 1,<br>280 / 1456	**19.2**	-
***Enterobacter* spp.**				
Aminoglycosides	**-**	n = 2,<br>1: 4 / 42,<br>2: 1114 / 20926	**5.3 – 9.5**	-
Carbapenems	**-**	n = 2,<br>1: 2 / 42,<br>2: 4884 / 55092	**4.8 – 8.9**	-
Cephalosporins (third generation)	**-**	n = 2,<br>1: 8 /24,<br>2: 13884 / 70048	**19.1 – 19.8**	-
Cephalosporins (fourth and fifth generation)	**-**	n = 2,<br>1: 5 / 42,<br>2: 3198 / 32161	**9.9 – 11.9**	-
Cotrimoxazole	**-**	n = 2,<br>1: 5 /42,<br>2: 5099 / 102973	**5.0 – 11.9**	-
Fluoroquinolones	**-**	n = 2,<br>1: 5 / 42,<br>2: 3164 / 40144	**7.9 – 11.9**	-
Fosfomycin	**-**	n = 1,<br>19971 / 58212	**34.3**	-
Penicillins	**-**	n = 2,<br>1: 7 /42<br>2: 21461 / 65957	**16.7 – 32.5**	-

Penicillins + beta-lactamase-inhibitor	**-**	n = 2,<br>1: 6 / 42,<br>2: 20354 / 97967	**14.3 – 20.8**	-
Tetracyclines	**-**	n = 1,<br>7 / 42	**16.7**	-
Trimethoprim	**-**	n = 1,<br>1725 / 23070	**7.5**	-
**Pathogen and antibiotic resistance**	**Pooled resistance proportion in % (95% CI)**	**No. of studies, No. of resistant isolates / No. of total isolates**	**Range of study estimates in %, number of studies**	**Heterogeneity in % (95% CI, p-value)**
E. faecalis				
Aminoglycosides	**-**	n = 1,<br>167 / 995	**16.8**	-
Carbapenems	**-**	n = 1,<br>1: 48 / 48<br>2: 28 / 7763	**0.4 – 100.0**	-
Cephalosporins (first and second generation)	**-**	n = 1,<br>48 / 48	**100.0**	-
Cephalosporins (third generation)	**-**	n = 1,<br>48 / 48	**100.0**	-
Fluoroquinolones	**-**	n = 1,<br>1: 48 / 48<br>2: 1327 / 3327	**39.9 - 100**	-
Glycopeptides (Vancomycin)	**0.0**<br>(0.0 - 0.0)	n = 6,<br>9 / 9423	**0.0 – 0.1**	I^2 = 0.0 (0.0 - 74.6, p=0.8534)
Linezolid	**-**	n = 1,<br>11 / 7997	**0.1**	-
Metronidazole	**-**	n = 1,<br>48 / 48	**100.0**	-
Nitrofurantoin	**-**	n = 1,<br>19 / 3954	**0.5**	-
Penicillins	**-**	n = 1,<br>21 / 7041	**0.3**	-
Penicillins + beta-lactamase-inhibitor	**-**	n = 1,<br>4 / 48<br>9 / 3475	**0.3 – 8.3**	-
Tetracyclines	**-**	n =1,<br>1: 0 / 48<br>2: 10 / 6548	**0.0 – 0.2**	-
E. faecium				
Aminoglycosides	**-**	n = 1,<br>1285 / 3514	**36.6**	-
Carbapenems	**-**	n = 2,<br>1: 58 / 58<br>2: 6388 / 6877	**92.9 – 100**	-
Cephalosporins (first and second generation)	**-**	n =1,<br>58 / 58	**100.0**	-
Cephalosporins (third generation)	**-**	n = 1,<br>58 / 58	**100.0**	-
Daptomycin	**-**	n = 1,<br>8 / 48	**16.7**	-
Fluoroquinolones	**-**	n =2,<br>1: 58 / 28	**92.7 – 100**	-

		2: 4075 / 4397		
Glycopeptides (Vancomycin)	**28.2** (23.9 - 32.7)	n = 10, 3803 / 15035	**0.0 – 82.5**	I^2 = 93.9 (90.8 - 96.0, p<0.0001
Linezolid	**-**	n = 2, 1: 5 / 185 2: 44 / 7366	**0.6 – 2.7**	-
Metronidazole	**-**	n = 1, 58 / 58	**100**	**-**
Penicillins	**-**	n = 1, 5796 / 6250	**92.7**	**-**
Penicillins + beta-lactamase-inhibitor	**-**	n = 2, 1: 58 / 58 2: 5309 / 5770	**92.0 – 100**	**-**
Tetracyclines	**0.87** (0.00-3.87)	n = 3, 31 / 6149	**0.0 – 3.7**	I^2 = 82.8 (47.4 - 94.4, p=0.003)
***Enterococcus* spp.**				
Aminoglycosides	**-**	n = 2, 1: 81 / 86 2: 1452 / 4509	**32.2 – 94.2**	-
Carbapenems	**45.8** (10.5 – 83.7)	n = 4, 9371 / 14914	**5.8 – 97.5**	I^2 = 99.2 (98.8 - 99.4, p<0.0001)
Cephalosporins (first and second generation)	**-**	n =1, 152 / 158	**96.2**	-
Cephalosporins (third generation)	**-**	n = 1, 152 / 158	**96.2**	-
Cotrimoxazole	**-**	n = 1, 56 / 86	**65.1**	-
Daptomycin	**-**	n = 1, 8 / 48	**16.7**	-
Fluoroquinolones	**-**	n = 2, 1: 154 / 158 2: 5402 / 7724	**69.9 – 97.5**	-
Glycopeptides (vancomycin)	**15.8** (11.1 - 21.1)	n = 15, 4003 / 26321	**0.0 – 52.2**	I^2 = 98.6 (98.3 - 98.9%, p<0.0001)
Linezolid	**0.6** (0.0 - 3.2)	n = 3, 60 / 15578	**0.0 – 2.7**	I^2 = 78.7 (31.6 - 93.4%, p=0.0092)
Metronidazole	**-**	n = 1, 158 / 158	**100.0**	-
Penicillins	**30.3** (13.0 – 51.0)	n = 3, 5841 / 13407	**17.4 – 43.8**	I^2 = 93.5 (84.3 - 97.3, p<0.0001)
Penicillins + beta-lactamase-inhibitor	**38.8** (20.5 – 58.8)	n = 3, 5425 / 9519	**14.0 – 57.5**	I^2 = 96.5 (93.6 - 98.1, p<0.0001)
Tetracyclines	**0.7** (0.0 - 3.0)	n = 3, 41 / 12797	**0.0 – 3.7**	I^2 = 85.9 (58.8 - 95.1%, p=0.0008)

sTable 2 (continued).

Pathogen and antibiotic resistance	Pooled resistance proportion in % (95% CI)	No. of studies, No. of resistant isolates / No. of total isolates	Range of individual study estimates in %	Heterogeneity in % (95% CI, p-value)
E. coli				
Aminoglycosides	**7.2**<br>(5.1 – 9.7)	n = 9,<br>36556 / 515313	**0.0 – 18.2**	I^2 = 99.7 (99.7 - 99.8, p = 0)
Carbapenems	**0.0**<br>(0.0 - 0.0)	n = 10,<br>616 / 619515	**0.0 – 0.1**	I^2 = 0.0 (0.0 - 62.4, p=0.9868)
Cephalosporins (first and second generation)	**15.3**<br>(11.5 – 19.6)	n = 7,<br>153870 / 1110209	**9.3 – 78.3**	I^2 = 88.8 (79.4 - 93.9, p<0.0001)
Cephalosporins (third generation)	**11.1**<br>(9.9 – 12.4)	n = 13,<br>133059 / 1464011	**7.1 – 19.2**	I^2 = 99.2 (99.0 - 99.3, p<0.0001)
Cephalosporins (fourth and fifth generation)	**-**	n = 2,<br>1 : 72 / 973,<br>2 : 40347 / 485181	**7.4 – 8.3**	-
Colistin	**-**	n = 1,<br>0 / 23	**0.0**	-
Cotrimoxazole	**26.2**<br>(22.6 – 30.1)	n = 10,<br>257269 / 1231832	**17.4 – 36.8**	I^2 = 93.3 (89.6 - 95.6, p<0.0001)
Fluoroquinolones	**21.3**<br>(19.9 – 22.8)	n = 12,<br>127773 / 648820	**15.1 – 30.0**	I^2 = 98.4 (97.9 - 98.7, p<0.0001)
Fosfomycin	**0.7**<br>(0.7 – 0.8)	n = 6,<br>11497 / 989242	**0.0 – 1.8**	I^2 = 0.0 (0.0 - 74.6, p=0.8401)
Glycopeptides (Vancomycin)	**-**	n = 1,<br>138 / 144	**95.8**	**-**
3/4 MDR	**-**	n = 2,<br>1: 2 / 28<br>2: 11 /65	**5.3 – 16.9**	**-**
Metronidazole	**-**	n = 1<br>144 / 144	**100.0**	**-**
Nitrofurantoin	**2.0**<br>(0.9 – 3.5)	n = 4,<br>10962 / 954061	**1.1 – 4.4**	I^2 = 81.2 (50.8 - 92.8, p=0.0012)
Penicillins	**51.2**<br>(48.0 – 54.5)	n = 11,<br>427891 / 976635	**39.7 – 87.0**	I^2 = 99.7 (99.7 - 99.8, p=0)
Penicillins + beta-lactamase-inhibitor	**30.8**<br>(25.5 – 36.4)	n = 10,<br>264338 / 862221	**12.3 – 73.9**	I^2 = 93.8 (90.5 - 95.9, p<0.0001)
Tetracyclines	**9.0**<br>(0.0 – 31.5)	n = 5,<br>6682 / 427174	**0.0 – 38.8**	I^2 = 99.6 (99.5 - 99.7, p<0.0001)
Trimethoprim	**25.2**<br>(20.1 – 30.7)	n = 3,<br>121101 / 524383	**20.4 – 36.4**	I^2 = 87.9 (66.2 - 95.7, p=0.0003)

sTable 2 (continued).

Pathogen and antibiotic resistance	Pooled resistance proportion in % (95% CI)	No. of studies, No. of resistant isolates / No. of total isolates	Range of individual study estimates in %	Heterogeneity in % (95% CI, p-value)
H. influenzae				
**Carbapenems**	**-**	n = 1,<br>64 / 474	**13.5**	**-**
**Penicillins**	**-**	n = 1,<br>533 / 2432	**21.9**	**-**
**Cephalosporins (third generation)**	**-**	n = 1,<br>27 / 2432	**1.1**	**-**
K. pneumoniae				
**Aminoglycosides**	**-**	n = 2,<br>1: 33 / 270<br>2: 4626 / 43820	**10.6 – 12.2**	-
**Carbapenems**	**1.7**<br>(0.0 – 5.9)	n = 4,<br>10768 / 286029	**0.7 – 6.3**	I^2 = 100.0%,<br>p = 0
**Cephalosporins (first and second generation)**	**-**	n = 1,<br>33235 / 221503	**15.0**	-
**Cephalosporins (third generation)**	**10.7**<br>(7.5 – 14.4)	n = 3,<br>22612 / 226825	**7.4 – 14.3**	I^2 = 96.2 (91.9 - 98.2, p<0.0001)
**Cephalosporins (fourth and fifth generation)**	**-**	n = 1,<br>8446 / 87373	**9.7**	-
**Cotrimoxazole**	**-**	n = 2,<br>1: 39 / 270<br>2: 26656 / 238992	**11.2 – 14.4**	-
**Fluoroquinolones**	**15.5**<br>(14.1 – 17.0)	n = 3,<br>13509 / 82486	**13.3 – 16.4**	I^2 = 69.2 (0.0 - 91.0, p=0.0388)
**Fosfomycin**	**-**	n = 2,<br>1: 12 / 92<br>2: 33850 / 169973	**13.0 – 19.9**	-
**Penicillins + beta-lactamase-inhibitor**	**-**	n = 1,<br>29875 / 150327	**19.9**	-
**Trimethoprim**	**-**	n = 1,<br>11522 / 81235	**14.2**	-
N. gonorrhoeae				
**Cephalosporins (third generation)**	**-**	n = 1,<br>9 / 468	**1.9**	-
**Macrolides**	**-**	n = 2,<br>1: 78 / 1404<br>2: 58 / 145	**5.6 – 40.0**	-

sTable 2 (continued).

Pathogen and antibiotic resistance	Pooled resistance proportion in % (95% CI)	No. of studies, No. of resistant isolates / No. of total isolates	Range of individual study estimates in %	Heterogeneity in % (95% CI, p-value)
P. aeruginosa				
Amikacin	**-**	n = 1,<br>28 / 985	**2.8**	-
Aminoglycosides	**4.9**<br>(4.4 – 5.4)	n = 4,<br>7807 / 150605	**2.3 – 5.9**	I^2 = 8.6 (0.0 - 86.0, p<0.3501)
Carbapenems	**17.0**<br>(11.9 – 22.8)	n = 6,<br>25922 / 201279	**12.8 – 25.1**	I^2 = 96.1 (93.7 - 97.6, p<0.0001)
Cephalosporins (third generation)	**10.1**<br>(6.6 - 14.2)	n = 5,<br>311 / 2515	**4.7 – 15.5**	I^2 = 81.5 (57.2 – 92.0, p=0.0002)
Cephalosporins (fourth and fifth generation)	**-**	n = 2,<br>1: 0 / 51<br>2: 142 / 985	**0.0 – 14.4**	-
Colistin	**-**	n = 1,<br>16 / 985	**1.6**	-
Fluoroquinolones	**24.9**<br>(19.3 – 30.9)	n = 6,<br>25598 / 85825	**15.8 – 33.3**	I^2 = 95.3 (92.2 - 97.2, p<0.0001)
MDR	**19.6**<br>(1.7 - 48.2)	n = 3,<br>24 / 190	**5.5 – 54.5**	I^2 = 92.4 (81.0 - 97.0, p<0.0001)
Penicillins	**-**	n = 2,<br>1: 6 / 51<br>2: 24801 / 182269	**11.8 – 13.6**	-
Penicillins + beta-lactamase-inhibitor	**12.6**<br>(9.0 – 16.7)	n = 5,<br>21553 / 205644	**5.9 – 21.9**	I^2 = 86.1 (69.7 - 93.7, p<0.0001)
***Salmonella* spp.**				
Carbapenems	**-**	n = 1,<br>0 / 11730	**0.0**	-
Cephalosporins (third generation)	**-**	n = 1,<br>206 / 13882	**1.5**	-

sTable 2 (continued).

Pathogen and antibiotic resistance	Pooled resistance proportion in % (95% CI)	No. of studies, No. of resistant isolates / No. of total isolates	Range of individual study estimates in %	Heterogeneity in % (95% CI, p-value)
S. aureus				
Aminoglycosides	**3.3** (2.0 - 4.9)	n = 3, 3710 / 85179	**2.3 - 4.4**	I^2 = 86.9 (62.6 - 95.4, p=0.0005)
Carbapenems	**2.5** (0.0 – 12.9)	n = 3, 21775 / 283686	**0.0 - 7.7**,	I^2 = 99.3 (98.8 - 99.5, p<0.0001)
Cephalosporins (first and second generation)	**2.5** (0.0 - 10.4)	n = 4, 14883 / 196288	**0.0 - 7.6**	I^2 = 98.9 (98.3 - 99.3, p<0.0001)
Cephalosporins (third generation)	**8.9** (0.0 – 29.8)	n = 3, 40375 / 158710	**3.2 - 25.5**	I^2 = 98.5 (97.3 - 99.1, p<0.0001)
Cephalosporins (fourth and fifth generation)	**-**	n = 1, 2013 / 33239	**6.1**	-
Clindamycin	**14.7** (11.1 – 18.7)	n = 4, 77124 / 477738	**9.7 - 20.2**	I^2 = 92.4 (83.7 – 96.4, p<0.0001)
Cotrimoxazole	**3.2** (1.5 - 5.6)	n = 5, 7549 / 487174	**0.7 - 13.2**	I^2 = 99.4 (99.2 - 99.5, p<0.0001)
Daptomycin	**-**	n = 1, 1 / 138	**0.7**	-
Fluoroquinolones	**14.7** (13.0 – 16.5)	n = 7, 45730 / 283772	**6.5 - 20.1**	I^2 = 81.5 (62.9 - 90.8, p<0.0001)
Fosfomycin	**0.5** (0.2 - 0.9)	n = 3, 10 / 1756	**0.0 - 0.7**	I^2 = 0 (0.0 - 89.6, p=0.4602)
Fusidic acid	**-**	n = 3, 1: 24 / 974 2: 21 / 589	**2.5 - 3.6**	-
Glycopeptides (vancomycin)	**0** (0.0 - 0.0)	n = 4, 0 / 416849	**0.0 - 0.0**	I^2 = 0.0 (0.0 - 84.7, p=0.4195)
Macrolides	**16.7** (12.5 - 21.3)	n = 4, 50795 / 294618	**10.2 - 20.7**	I^2 = 93.9 (86.9 - 96.9, p<0.0001)
MRSA (beta-lactamase stable penicillins)	**7.9** (5.2 – 11.0)	n = 16, 18422 / 285472	**0.0 - 63.2**	I^2 = 98.5 (98.2 - 98.8, p<0.0001)
Mupirocin	**-**	n = 2, 1: 2 / 965 2: 0 / 483	**0.0 - 0.2**	-
Nitrofurantoin	**-**	n = 1, 31051 / 97710	**31.8** n = 1	-
Penicillins	**70.7** (65.8 – 75.4)	n = 4, 294350 / 391355	**62.3 - 75.2**	I^2 = 83.1% (56.8 - 93.4), p=0.0005
Penicillins + beta-lactamase-inhibitor	**2.7** (0.0 - 10.8)	n = 4, 16427 / 211426	**0.0 - 7.8**	I^2 = 98.9 (98.4 - 99.3, p<0.0001)
Rifampicin	**0.2** (0.1 - 0.3)	n = 5, 1083 / 358688	**0.0 - 0.8**	I^2 = 9.6 (0.0 – 81.2, p=0.3518)
Tetracyclines	**5.9** (3.4 – 9.0)	n = 4, 12684 / 296399	**2.7 - 9.7**	I^2 = 93.9 (87.5 – 97.0, p<0.0001)
Trimethoprim	**-**	n = 1, 207 / 9305	**2.2**	-

sTable 2 (continued).

Pathogen and antibiotic resistance	Pooled resistance proportion in % (95% CI)	No. of studies, No. of resistant isolates / No. of total isolates	Range of individual study estimates in %	Heterogeneity in % (95% CI, p-value)
S. pneumoniae				
Carbapenems	**-**	n = 2,<br>1: 0 / 34<br>2: 12 / 5174	**0.0 - 0.2**	-
Cephalosporins (first and second generation)	**-**	n = 2,<br>1 : 1 / 34<br>2: 129 / 10878	**1.2 - 2.9**	-
Cephalosporins (third generation)	**-**	n = 2,<br>1: 0 / 34<br>2: 27 / 13865	**0.0 - 0.2**	-
Cephalosporins (fourth and fifth generation)	**-**	n = 1,<br>3 / 1693	**0.2**	-
Clindamycin	**-**	n = 1,<br>1 / 18127	**6.7**	-
Cotrimoxazole	**-**	n = 1,<br>1305 / 16766	**7.8**	-
Fluoroquinolones	**-**	n = 2,<br>1: 0 / 34<br>2: 83 7 18752	**0.0 - 0.4**	-
Glycopeptides (vancomycin)	**-**	n = 2,<br>1: 0 / 34<br>2: 0 / 15196	**0.0 - 0.0**	-
Linezolid	**-**	n = 2,<br>1: 0 / 34<br>2: 3 / 9193	**0.0 - 0.03**	-
Macrolides	**-**	n = 1,<br>1414 / 13612	**10.4**	-
MDR	**-**	n = 1,<br>7 / 182	**3.9**	-
Penicillins	**-**	n = 2,<br>1: 1 / 34<br>2: 315 / 20415	**1.5 - 2.9**,	-
Penicillins + beta-lactamase-inhibitor	**-**	n = 2,<br>1: 1 / 34<br>2: 72 / 8382	**0.9 - 2.9**	-
Rifampicin	**-**	n = 1,<br>2 / 6525	**0.03**	-
Tetracyclines	**-**	n = 1,<br>1102 / 12030	**9.2**	-

7.2 Subgroup-analysis: Outpatients vs. Inpatients

sTable 3: Analysis of ARS data from 2014-2020.

Pathogen and antibiotic resistance	Mean resistance proportion among inpatients in %	Mean resistance proportion among outpatients in %	Odds Ratio (95% CI, adjusted p-value)
S. aureus			
Oxacillin (MRSA)	12.0	7.3	0.59 (0.58 – 0.6, p < 0.0001)
E. faecium			
Vancomycin (VRE)[2]	19.6	15.5[3]	0.71 (0.57 – 0.88, p = 0.0304)
A. baumannii (complex)			
Carbapenems (meropenem)	4.1	1.4	0.33 (0.30 – 0.37, p < 0.0001)
Fluoroquinolones (ciprofloxacin)	8.6	4.9	0.54 (0.51 – 0.58, p < 0.0001)
K. pneumoniae			
Carbapenems (ertapenem)	1.1	0.4	0.35 (0.31 – 0.4, p < 0.0001)
TGC (ceftazidime)	11.7	7.7	0.63 (0.62 – 0.64, p < 0.0001)
Fluoroquinolones (moxifloxacin)	18.1	13.7	0.71 (0.7 – 0.73, p < 0.0001)
P. aeruginosa			
Carbapenems (imipenem)	14.1	9.2	0.62 (0.61 – 0.63, p < 0.0001)
TGC (ceftazidime)	9.4	4.8	0.49 (0.47 – 0.5, p < 0.0001)
Fluoroquinolones (levofloxacin)	21.0	21.2	1.0 (0.99 – 1.0, p = 1.0)
Enterobacter* spp. *(E. cloacae)			
Carbapenems (ertapenem)	8.7	3.3	0.35 (0.33 – 0.37, p < 0.0001)
TGC (ceftriaxon)	25.9	12.3	0.4 (0.39 – 0.41, p < 0.0001)
Fluoroquinolones (moxifloxacin)	9.1	6.4	0.7 (0.65 – 0.73, p < 0.0001)
E. coli			
Carbapenems (ertapenem)	0.14	0.07	0.57 (0.49 – 0.63, p < 0.0001)
TGC (cefotaxim)	11.3	7.5	0.64 (0.63 – 0.65, p < 0.0001)
Fluoroquinolones (moxifloxacin)	22.7	18.9	0.79 (0.78 – 8.0, p < 0.0001)

[2] Bloodstream-isolates

[3]ARS-data from 2015-2020 (outpatient data not reported for 2014)

sFig. 1. Mean resistance proportions of ESKAPE-E pathogens among inpatients and outpatients in Germany based on ARS-Data from 2014-2020

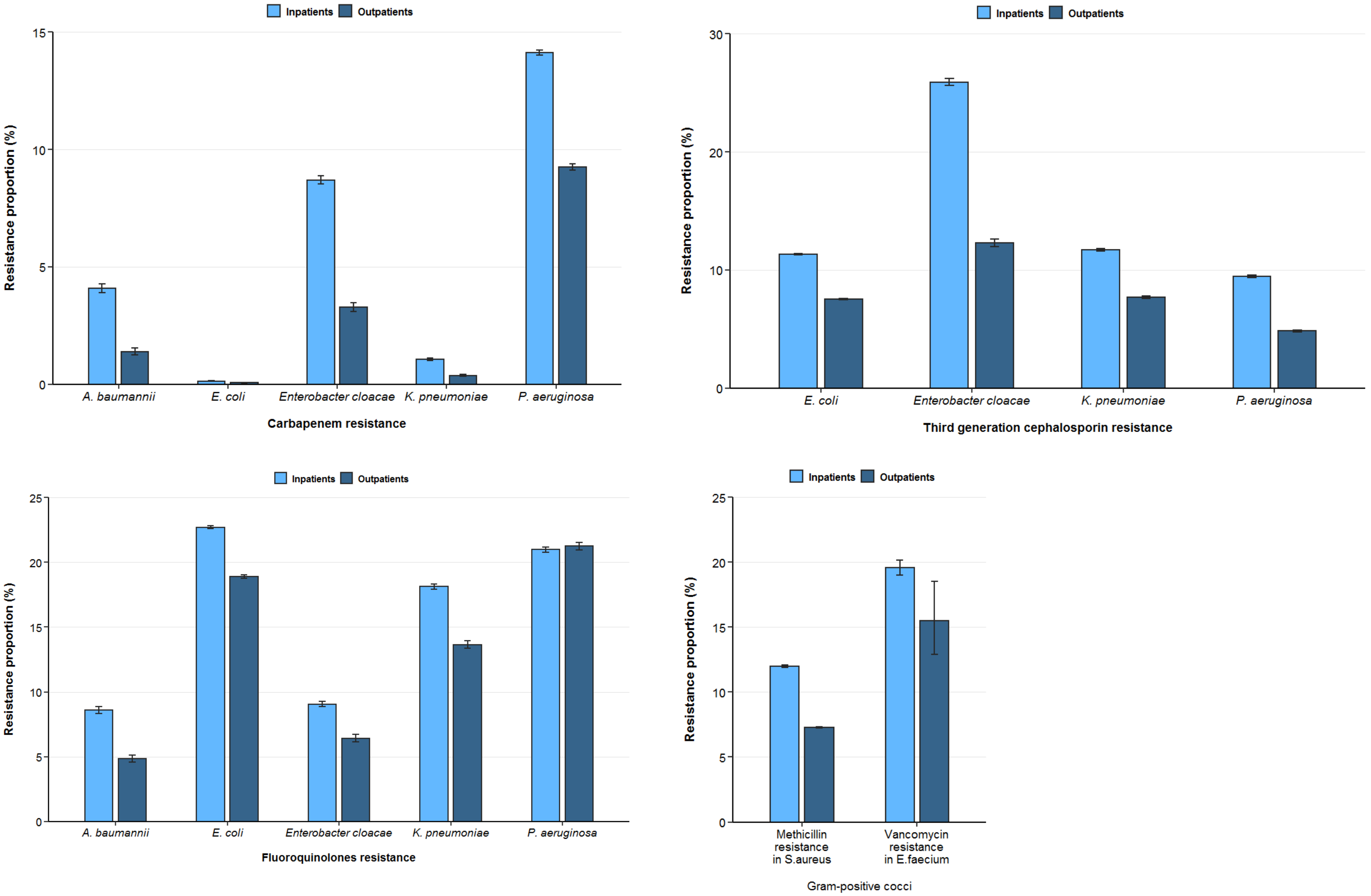

7.3 Time trend analysis

Graphic representation of the time trend of resistance proportions from inpatients and outpatients based on ARS-Data from 2014-2020.

sFig. 2. Vancomycin resistance proportions in *E. faecium* (VRE) from 2014-2020 in Germany (Blood culture isolates)

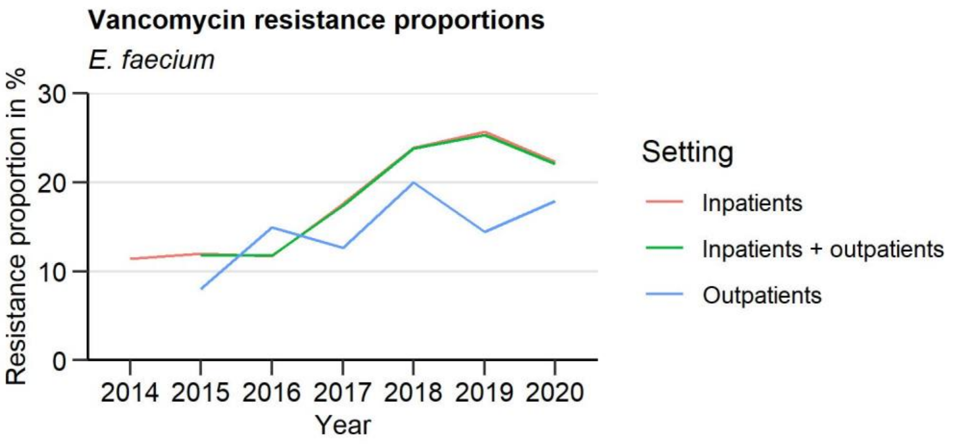

sFig. 3. Oxacillin resistance proportions in *Staphylococcus aureus* (MRSA) from 2014-2020 in Germany

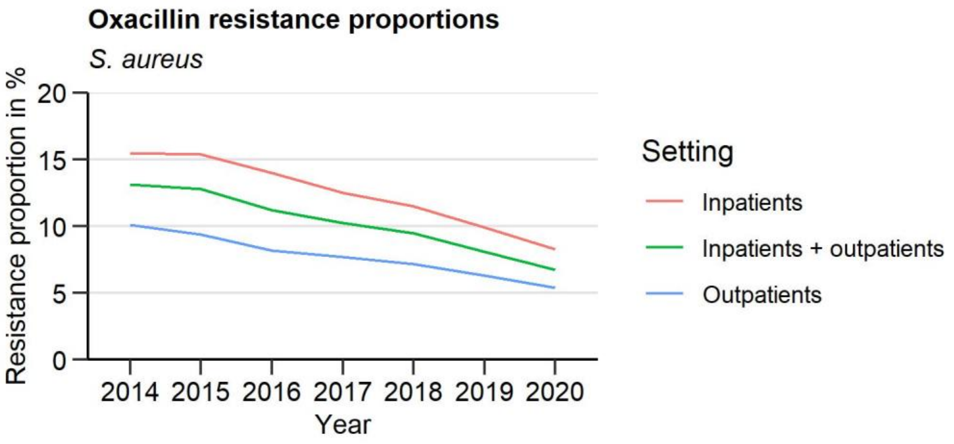

sFig. 4. Carbapenem (A), third generation cephalosporin (B) and fluoroquinolone (C) resistance proportions in *K. pneumoniae* from 2014-2020 in Germany

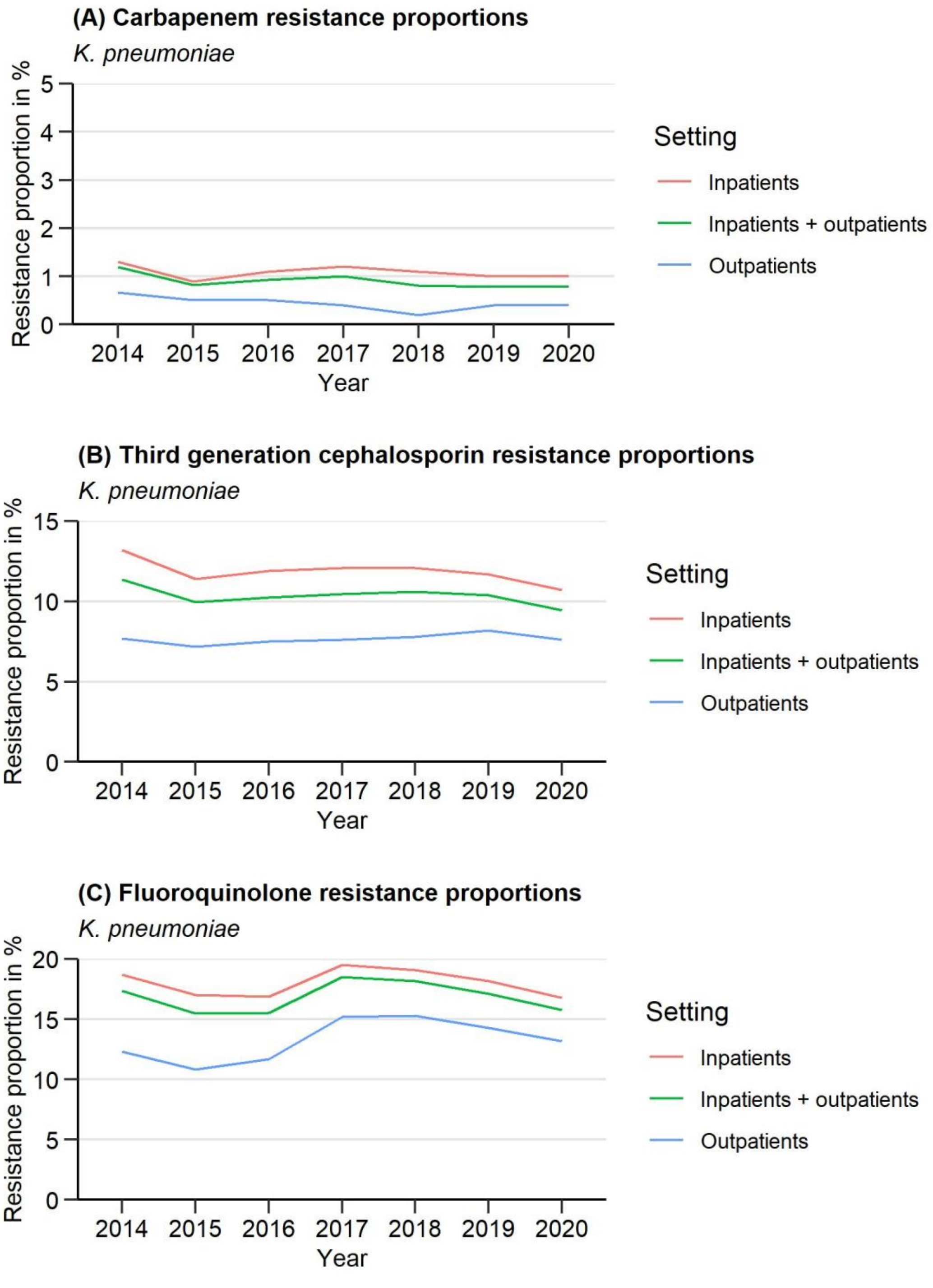

sFig. 5. Carbapenem (A) and fluorquinolone (B) resistance proportions in *A. baumannii complex* from 2014-2020 in Germany

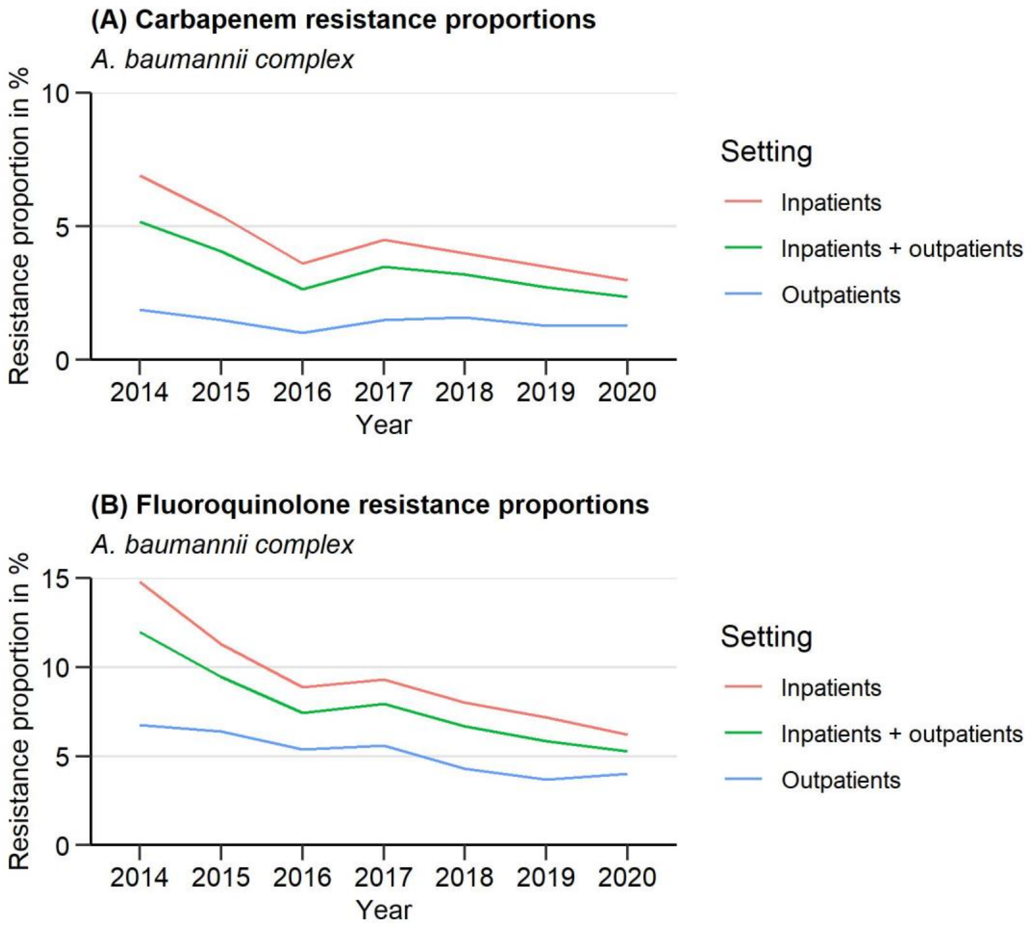

sFig. 6. Carbapenem (A), third generation cephalosporin (B) and fluorquinolone (C) resistance proportions in *P. aeruginosa* from 2014-2020 in Germany

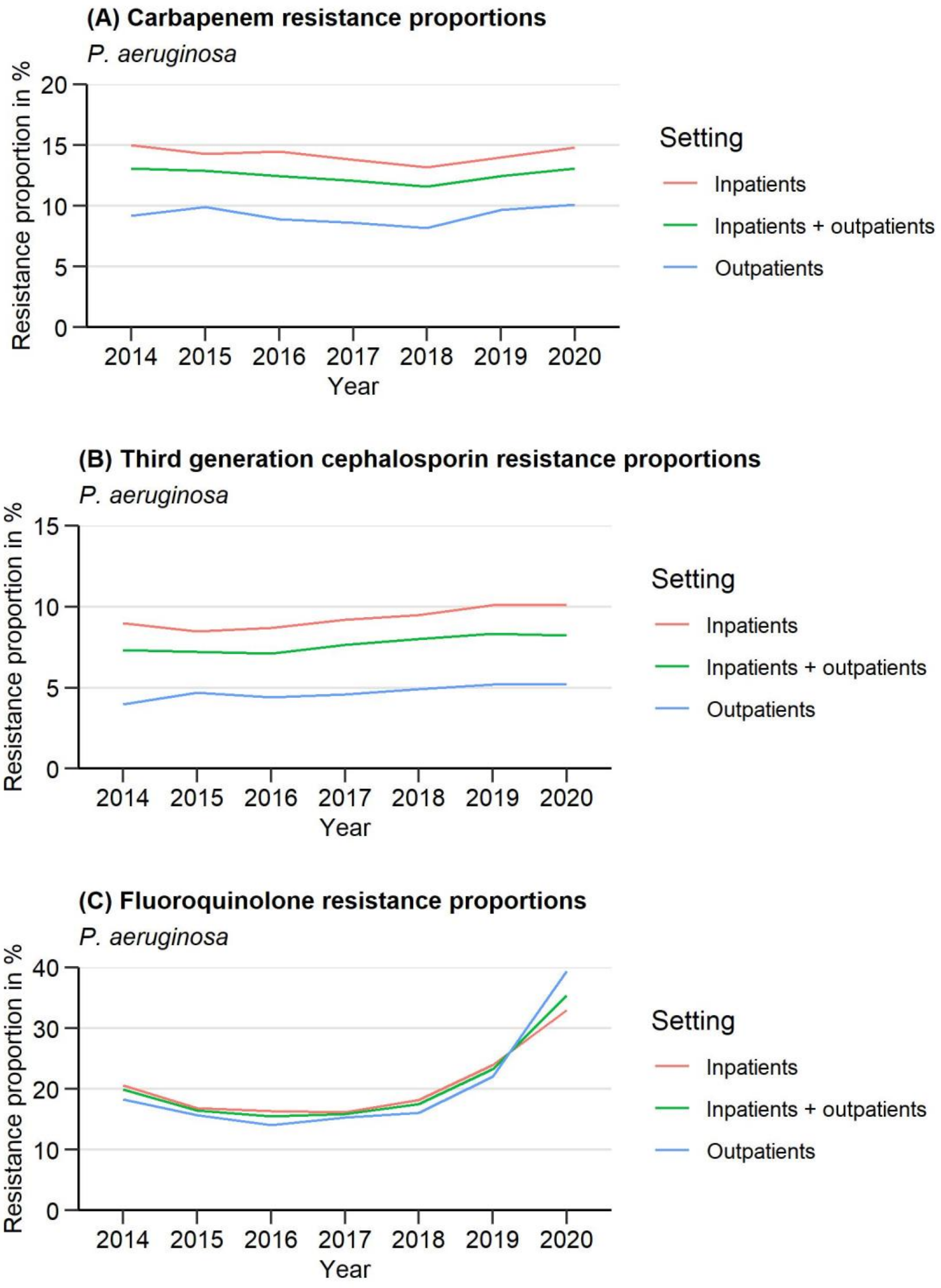

sFig. 7. Carbapenem (A), third generation cephalosporin (B) and fluorquinolone (C) resistance proportions in *E. cloacae* from 2014-2020 in Germany

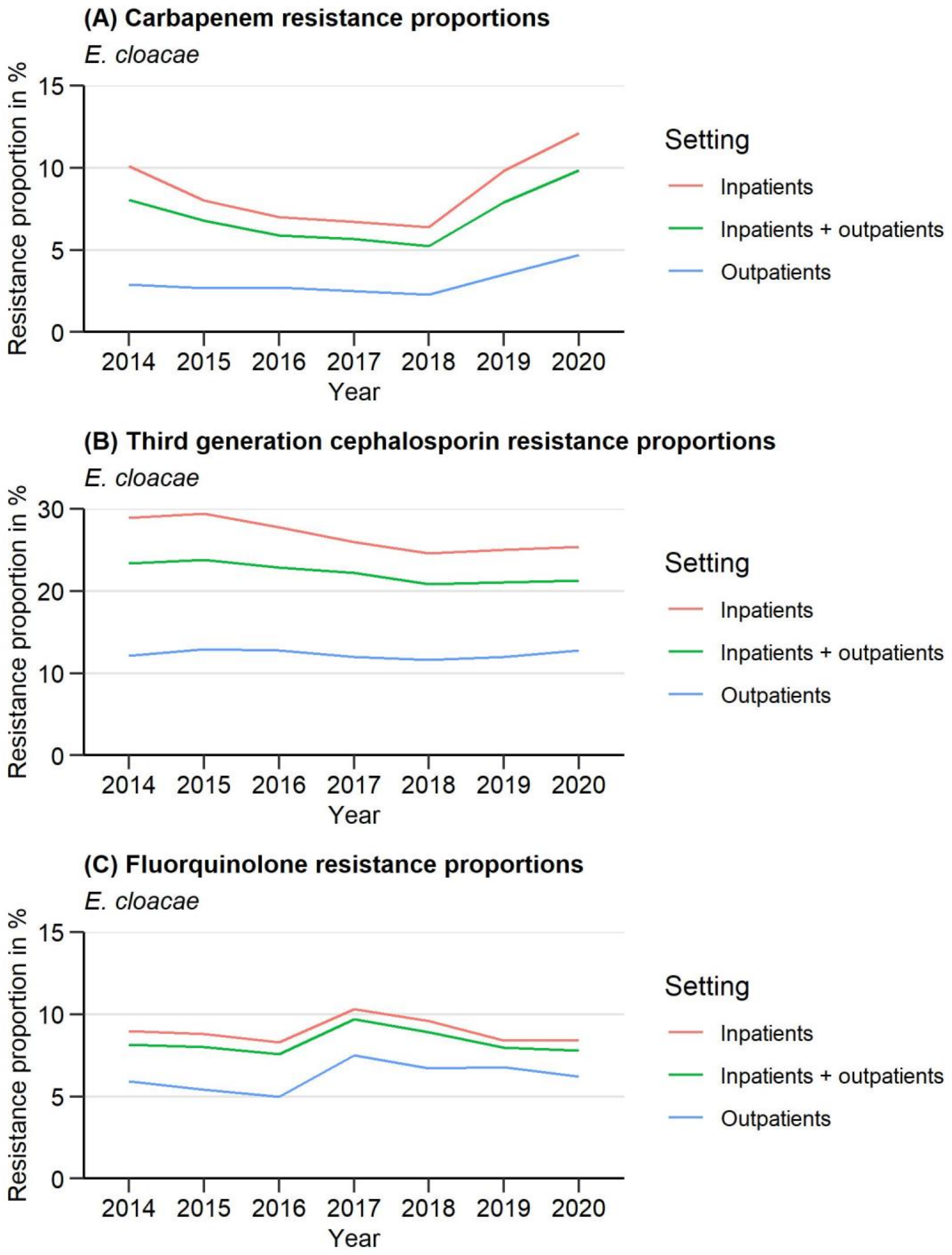

sFig. 8. Carbapenem (A), third generation cephalosporin (B) and fluorquinolone (C) resistance proportions in *E. coli* from 2014-2020 in Germany

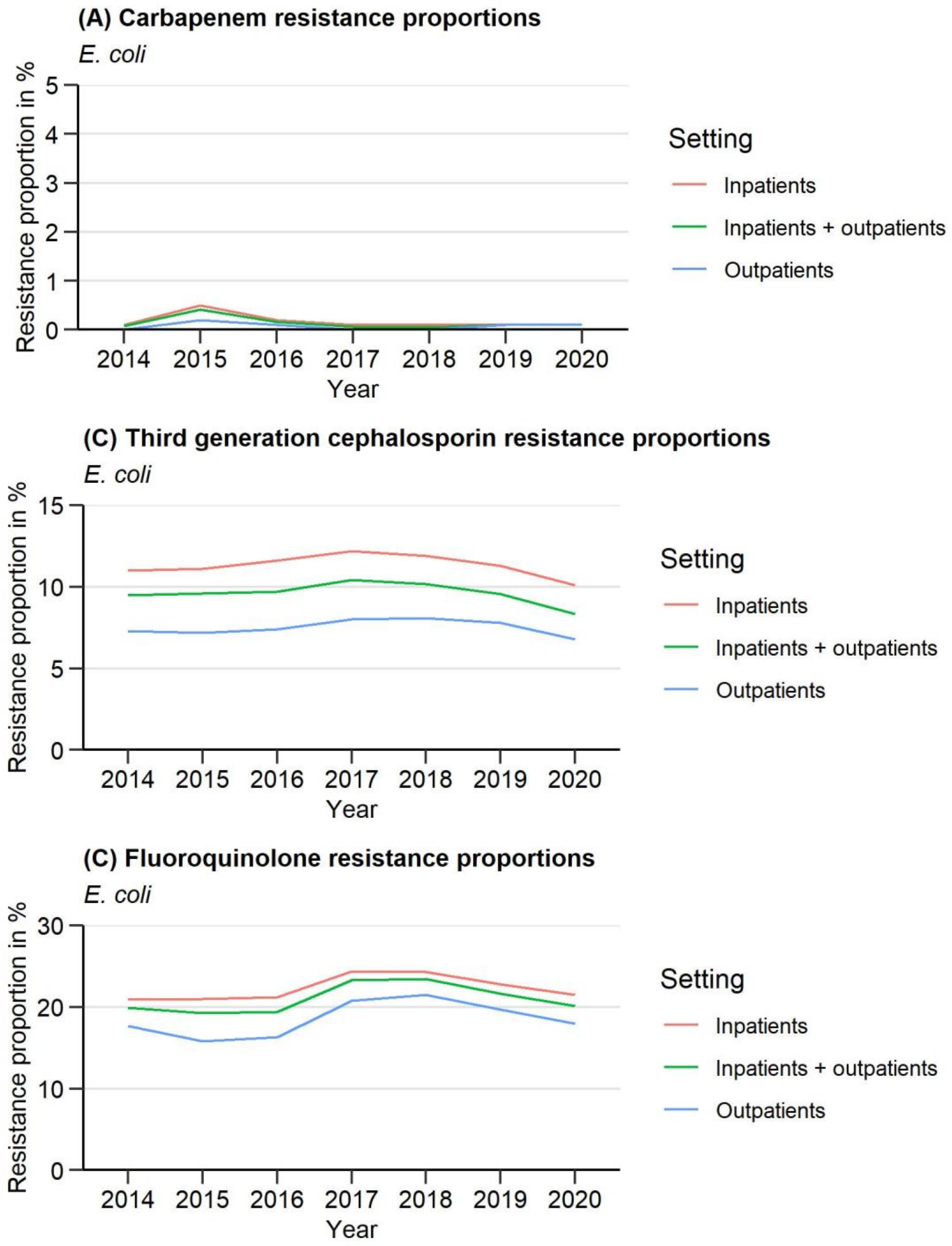

7.4 AMR proportions in different countries

sFig.9: Comparison of resistance proportions in ESKAPE-E pathogens between Germany and other countries

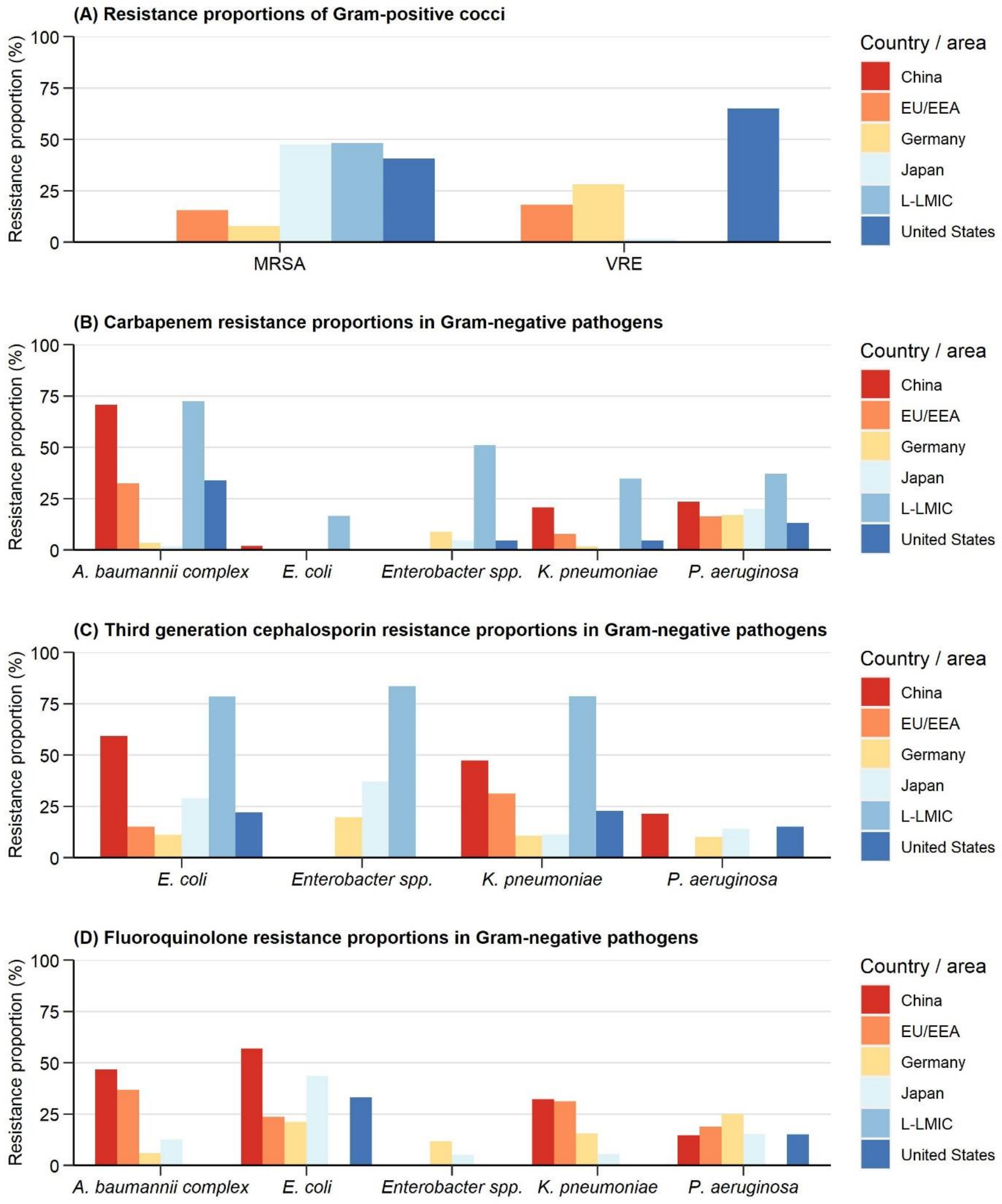

Abbreviations: L-LMIC = Low-/lower-middle income countries; EU/EEA = Europe and European Economic Area. For detailed information on data sources see Table 2.

7.5 Case fatality rate

sTable 4: Pooled proportions of all-cause and attributable case fatality rates in infections with antibiotic resistant pathogens in Germany

Pathogen	Pooled proportion in % (95% CI)	No. of studies, No. of deaths / No. total patients	Number of studies, range of study estimates in %	Heterogenity in % (95% CI), p-value
All-cause case fatality rate				
VRE (*E. faecium)*	**32.4** (17.9 – 48.8)	n = 4, 91 / 244	15.4 - 50.5	I^2 = 84.6 (61.5-93.8), p=0.0002
VRE (*Enterococcus* spp.)	**31.8** (21.9 – 42.6)	n = 7, 160 / 503	15.4 - 50.5	I^2 = 78 (54.3-89.4), p=0.0001
MRSA	**28.5** (20.0 – 37.9)	n = 8, 944 / 5428	12.4 - 42.9	I^2 = 96.7 (95.0-97.7), p<0.0001
ESBL *E. coli*	**-**	n = 2, 1: 45 / 178, 2: 38 / 160	**23.8 - 25.3**	-
Non-ESBL *E. coli*	**-**	n = 1, 233 / 1321	**17.6**	-
ESBL *K. pneumoniae*	**-**	n = 2, 1: 16 / 66, 2: 16 /59	**24.2 – 27.1**	-
Non-ESBL *K. pneumoniae*	**-**	n = 1, 72 / 286	**25.2**	-
3/4 MDR *P. aeruginosa*	**-**	n = 2, 1: 19 / 45, 2: 17 / 27	**42.2 – 63.0**	-
Non-3/4 MDR *P. aeruginosa*	**-**	n = 2, 1: 19 / 42, 2: 26 / 86	**30.2 – 45.2**	-
Attributable case fatality rate (as defined by the study authors)				
VRE	**-**	n = 2, 1: 11 / 232, 2: 10 / 47	n = 2, **4.7 – 17.0**	-
MRSA	**8.1** (3.7 – 13.7)	n = 5, 313 / 4140	7.0 - 33.3	I^2 = 77.9 (47-90.8), p=0.0012

7.6 Subgroup analyses of antimicrobial resistance proportions: studies with national data vs. studies with regional data.

sTable 5: Subgroup analyses of antimicrobial resistance proportions: studies with national data *vs.* studies with regional data.

Pathogen	Antibiotic resistance	No. of studies with national data	No. of studies with regional data	subgroup analyses in meta-analysis: p-value (adjusted p-value[a])
E. faecium	Glycopeptides (vancomycin)	n = 3	n = 7	0.6930 (0.8085)
Enterococcus spp.	Glycopeptides (vancomycin)	n = 6	n = 9	0.8862 (0.8862)
E. coli	Third-generation cephalosporins	n = 4	n = 9	0.6907 (0.8085)
E. coli	Fluoroquinolones	n = 3	n = 9	0.2024 (0.7084)
P. aeruginosa	Carbapenems	n = 3	n = 3	0.4359 (0.8085)
P. aeruginosa	Fluoroquinolones	n = 3	n = 3	0.5056 (0.8085)
S. aureus	methicillin (beta-lactamase stable penicillins)	n = 5	n = 11	0.0289 (0.2023)

[a] P-values were adjusted for multiple comparisons using the Benjamini & Hochberg method (10)

Subgroup analyses were only performed when each subgroup had at least three studies.

National data: Studies included isolates/patients from all or most federal states of Germany.

Regional data: Studies included isolates/patients from one or few selected cities/federal states.

8. References of the supplementary material

1. Ouzzani M, Hammady H, Fedorowicz Z, Elmagarmid A. Rayyan-a web and mobile app for systematic reviews. Systematic reviews. 2016;5(1):210.
2. CDC/NHSN. Surveillance Definitions for Specific Types of Infections 2022 [updated Jan. Available from: https://www.cdc.gov/nhsn/pdfs/pscmanual/17pscnosinfdef_current.pdf
3. Robert Koch-Institut. ARS: Teilnahme - Reichweite - Repräsentativität 2022 [Available from: https://ars.rki.de/Content/Project/Participation.aspx.
4. R Core Team. R: A language and environment for statistical computing: R Foundation for Statistical Computing; 2022 [R version 4.1.2 (2021-11-01):[Available from: https://www.r-project.org/.
5. Schwarzer G, Carpenter JR, Rücker G. Meta-Analysis with R: Springer International Publishing Switzerland; 2015.
6. Schwarzer G. meta: An R package for meta-analysis. R News. 2007;7/3:40 ff.
7. Curtin F, Schulz P. Multiple correlations and Bonferroni's correction. Biological psychiatry. 1998;44(8):775-7.
8. Hoy D, Brooks P, Woolf A, Blyth F, March L, Bain C, et al. Assessing risk of bias in prevalence studies: modification of an existing tool and evidence of interrater agreement. Journal of clinical epidemiology. 2012;65(9):934-9.
9. Wells G. SB, O'Connell D, Peterson J, Welch V, Losos M, et al. The Newcastle-Ottawa Scale (NOS) for assessing the quality of nonrandomised studies in meta-analyses Internet: University of Ottawa; 2014 [Available from: http://www.ohri.ca/programs/clinical_epidemiology/oxford.asp.
10. Benjamini Y, Hochberg Y. Controlling the False Discovery Rate: A Practical and Powerful Approach to Multiple Testing. Journal of the Royal Statistical Society: Series B (Methodological). 1995;57(1):289-300.
